# LMFE: A Novel Method for Predicting Plant LncRNA Based on Multi-Feature Fusion and Ensemble Learning

**DOI:** 10.3390/genes16040424

**Published:** 2025-03-31

**Authors:** Hongwei Zhang, Yan Shi, Yapeng Wang, Xu Yang, Kefeng Li, Sio-Kei Im, Yu Han

**Affiliations:** 1 Faculty of Applied Sciences, Macao Polytechnic University, Macau SAR 999074, China; hongwei.zhang@mpu.edu.mo (H.Z.); xuyang@mpu.edu.mo (X.Y.); marcusim@mpu.edu.mo (S.-K.I.); kefengl@mpu.edu.mo (K.L.); 2 State Key Laboratory of Networking and Switching Technology, Beijing University of Posts and Telecommunications, Beijing 100876, China; 3 Faculty of Civil Engineering, Southwest Forestry University, Kunming 650224, China; hanyu@swfu.edu.cn

**Keywords:** plant lncRNA prediction, multi-feature fusion, ensemble learning, cross-species, LMFE

## Abstract

**Background/Objectives**: Long non-coding RNAs (lncRNAs) play a crucial regulatory role in plant trait expression and disease management, making their accurate prediction a key research focus for guiding biological experiments. While extensive studies have been conducted on animals and humans, plant lncRNA research remains relatively limited due to various challenges, such as data scarcity and genomic complexity. This study aims to bridge this gap by developing an effective computational method for predicting plant lncRNAs, specifically by classifying transcribed RNA sequences as lncRNAs or mRNAs using multi-feature analysis. **Methods**: We propose the lncRNA multi-feature-fusion ensemble learning (LMFE) approach, a novel method that integrates 100-dimensional features from RNA biological properties-based, sequence-based, and structure-based features, employing the XGBoost ensemble learning algorithm for prediction. To address unbalanced datasets, we implemented the synthetic minority oversampling technique (SMOTE). LMFE was validated across benchmark datasets, cross-species datasets, unbalanced datasets, and independent datasets. **Results**: LMFE achieved an accuracy of 99.42%, an F1_score_ of 0.99, and an MCC of 0.98 on the benchmark dataset, with robust cross-species performance (accuracy ranging from 89.30% to 99.81%). On unbalanced datasets, LMFE attained an average accuracy of 99.41%, representing a 12.29% improvement over traditional methods without SMOTE (average ACC of 87.12%). Compared to state-of-the-art methods, such as CPC2 and PLEKv2, LMFE consistently outperformed them across multiple metrics on independent datasets (with an accuracy ranging from 97.33% to 99.21%), with redundant features having minimal impact on performance. **Conclusions**: LMFE provides a highly accurate and generalizable solution for plant lncRNA prediction, outperforming existing methods through multi-feature fusion and ensemble learning while demonstrating robustness to redundant features. Despite its effectiveness, variations in performance across species highlight the necessity for future improvements in managing diverse plant genomes. This method represents a valuable tool for advancing plant lncRNA research and guiding biological experiments.

## 1. Introduction

The rapid advancement of high-throughput sequencing technology has led to the generation of substantial omics data, such as genomics, transcriptomics, and proteomics data. The analysis of these omics data enables the elucidation of specific biological phenomena at a microscopic level, rendering omics data research a current hotspot in biology. The discipline of computational biology, which employs computational methods to mine and analyze biological data, has enabled valuable biological insights and offers a convenient and efficient approach to the analysis of biological data [1]. For instance, recent studies have employed deep sequencing and bioinformatics analysis to identify regulatory networks, such as the lncRNA/circRNA–miRNA–mRNA network in nasopharyngeal carcinoma, underscoring the efficacy of computational approaches in uncovering complex biological interactions [2].

Ribonucleic acid (RNA) is the subject of transcriptomic research and is an important biomolecule. RNA can be classified into two categories based on its capacity to encode proteins, namely coding RNAs (cRNAs) and non-coding RNAs (ncRNAs) [3]. Specifically, cRNA refers to messenger RNAs (mRNAs), while ncRNA encompasses various types. Numerous prediction methods are available [4], based on the length of ncRNA sequences, and they are divided into two subclasses, namely small or short noncoding RNAs (sncRNAs, 18–200 nt) and lncRNAs (>200 nt) [5,6]. ncRNA constitutes over 90% of the RNA transcribed from the genome and has long been regarded as “junk” or “noise” in the genome [7]. However, with ongoing research, as a class of ncRNAs, lncRNAs have garnered significant attention due to their key roles in gene regulation, cellular processes, and plant development. For instance, the lncRNA H19 has been demonstrated to contribute to tumor growth and metastasis in breast cancer, and its silencing can reverse doxorubicin resistance by modulating apoptotic pathways [8]. In gastric cancer tissues, specific lncRNAs, such as HOTAIR, exhibit significant expression differences, and these differentially expressed lncRNAs may serve as potential prognostic indicators and therapeutic targets for gastric cancer [9].

In plants, lncRNAs have been demonstrated to possess biological functions. For instance, several lncRNAs have been identified, including ColdAIR and CoolAIR, which regulate gene expression and influence flowering time [10]. Beyond model plants, specialized metabolic pathways involving lncRNAs have been elucidated in the genomes of medicinal plants, underscoring their roles in phytochemical diversity and pharmacological potential [11]. Additionally, the identification of anthocyanin biosynthesis genes in rice pericarp using computational tools, such as PCAMP illustrates how transcriptomic data can link lncRNAs to economically important traits [12]. The identification of lncRNAs can be accomplished through laboratory-based experimental methods, including RNA immunoprecipitation (RIP), Northern blotting, and reverse transcription polymerase chain reaction (RT-PCR), which directly process plant samples to detect lncRNAs. Although these methods exhibit high accuracy (ACC), they are time-consuming and resource-intensive, rendering them unsuitable for large-scale applications, particularly when dealing with the substantial volume of data generated by high-throughput sequencing technology. Given the specificity and significance of lncRNA in plant biological processes, the accurate prediction of lncRNA has emerged as a primary concern among researchers. With the advancements in computational methods, in both theory and technology, and their successful application across various fields of bioinformatics, researchers have increasingly turned to computational methods to identify and explore lncRNAs, as well as to investigate their functional roles in biological processes. Numerous computational methods have been developed and designed for this research. However, enhancing the performance and accuracy of prediction methods, particularly in the recognizing lncRNA in plants, remains an ongoing challenge that demands resolution. Prediction refers to the computational classification transcribed RNA sequences as lncRNAs or mRNAs based on multi-feature analysis.

In this study, we introduce several innovations or improvements. First we present a well-designed lncRNA prediction method that outperforms previous studies. By considering both local and global features of lncRNA sequences, a set of low-dimensional yet informative features were extracted. This addresses the issue of low efficiency caused by the high dimensionality of feature vectors in previous studies. Second, we compared the predictive performance of shallow machine learning methods with ensemble learning methods, further confirming the effectiveness of ensemble learning in lncRNA prediction. Third, we validated the minimal impact of redundant features on the performance of LMFE. Fourth, we conducted comparative research experiments to evaluate the performance of our LMFE method across various plant species, which further confirmed its effectiveness in predicting lncRNAs across species.

The remainder of this paper is structured as follows. Section 2 reviews related works, offering an overview of existing methods and challenges in plant lncRNA prediction. Section 3 outlines the LMFE method, encompassing data processing, multi-feature fusion, the prediction process, and method evaluation. Section 4 presents results and analysis, including cross-species validations and comparisons with state-of-the-art methods. Section 5 concludes the study by summarizing key findings and discussing challenges and future directions in plant lncRNA prediction.

## 2. Related Works

The application of machine learning methods to the identification and analysis of lncRNAs has emerged as a challenging research area. For instance, Yu-Jian Kang et al. [13] developed a method called the Coding Potential Calculator (CPC2) to evaluate the protein-coding potential of transcripts. CPC2 extracts the length of open reading frame (ORF) features, GC content, and other biological parameters from known mRNAs and ncRNAs. By training the support vector machine (SVM) [14], the coding potential of transcripts can be effectively identified and predicted. Compared to the previous version of the CPC [15], the CPC2 has enhanced both performance and computational efficiency. PLEKv2 [16] is an innovative method for predicting lncRNAs and mRNAs based on intrinsic sequence features and coding network models. It is an enhanced version of the misalignment tool PLEK [17], featuring models specifically designed for animals and plants. The encoding network model integrates calibrated ORF length and features from multiple k-mer frequencies, utilizing convolutional neural network algorithms for sequence classification. PLEKv2 demonstrates high ACC in predicting human datasets, achieving an ACC rate of 98.7%, and it also performs well in cross-species predictions. SUN et al. proposed a method called the Coding-Non-Coding Index (CNCI) in their research [18], which classifies coding sequences from non-coding sequences by analyzing adjacent nucleotide triplets. The results indicate that this research has shown excellent performance in various aspects. Wang and Yin conducted a study on the feature relationships between lncRNA and cRNA sequences. They proposed a novel method for feature extraction based on the differences in ORF length and GC content between the two sequences, naming it LGC (ORF length and GC content). Experimental validation demonstrated the effectiveness of this method in identifying lncRNA across multiple species datasets [19]. Meng et al. proposed a tool named PlncRNA-Hdeep [20], which offers a new and effective tool for predicting plant lncRNAs. It utilizes a hybrid deep learning approach and diverse feature encoding, significantly enhancing prediction performance. Beyond transcriptomic analysis, computational methods have been extended to other plant-related applications, such as multispectral polarimetric bidirectional reflectance studies of plant canopies, which provide insights into structural and biochemical properties, offering complementary data for lncRNA-related phenotypic studies [21].

Although existing research on lncRNAs primarily focuses on animals and humans, studies on plant lncRNAs are relatively new and remain scarce, particularly in their identification. lncRNAs are widely present in the transcriptomes of both animals and plants, and the functional mechanisms of the majority of lncRNAs are not well understood. Existing methods used in animal transcriptomes cannot be directly applied to plant transcriptomes due to significant differences in the occurrence, evolution, and functionality of lncRNAs between animals and plants [22]. In recent years, deep learning methods have increasingly been employed in studies focused on identifying lncRNAs in plants. Further research is needed to explore methods for enhancing model performance.

## 3. Materials and Methods

This study investigates lncRNA prediction methods in plants and proposes a novel method called lncRNA multi-feature-fusion ensemble learning (LMFE). LMFE utilizes a fusion of multi-perspective, multi-feature RNA sequence encoding methods and employs ensemble learning for data training.

### 3.1. Overall Framework of LMFE

**Figure 1:**
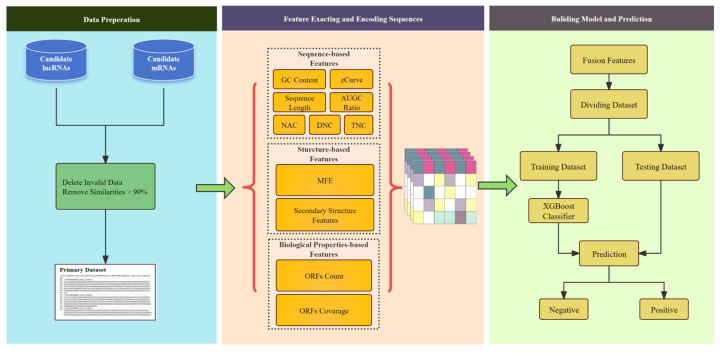
The overall framework of the LMFE consists of three steps. The first step involves the data preparation stage, where we obtained the lncRNA data as positive samples and the mRNA sequences as negative samples. In the second step, we focus on sequence representation and feature extraction, comprehensively capturing sequence features by considering the biological properties of RNA, sequence-based features, and structure-based features. Finally, we constructed an extreme gradient boosting (XGBoost) [23] based on ensemble learning to predict lncRNAs.

In this framework, we constructed a novel vector for effective multi-feature fusion with a reduced number of dimensions, comprehensively considering the structural and physicochemical features of the RNA sequences. The parameters of the ensemble learning method were optimized. The performance of the framework was validated using 5-fold cross-validation. As shown in Figure 1.

### 3.2. Datasets

In this study, we obtained lncRNA data in the FASTA format as positive samples from CANTATA (v3.0), and we downloaded mRNA data in the FASTA format as negative samples from EnsemblPlants (v59). The benchmark data we obtained included the following ten species: *Arabidopsis thaliana* (*A. thaliana*), *Vigna radiata* (*V. radiata*), *Zea mays* (*Z. mays*), *Sorghum bicolor* (*S. bicolor*), *Oryza sativa* (*O. sativa*), *Populus trichocarpa* (*P. trichocarpa*), *Selaginella moellendorffii* (*S. moellendorffii*), *Galdieria sulphuraria* (*G. sulphuraria*), *Triticum aestivum* (*T. aestivum*), and *Solanum lycopersicum* (*S. lycopersicum*). Additionally, an independent test dataset contained the following six species: *Vigna angularis* (*V. angularis*), *Sesamum indicum* (*S. indicum*), *Brachypodium distachyon* (*B. distachyon*), *Musa acuminata* (*M. acuminata*), *Marchantia polymorpha* (*M. polymorpha*), and *Nymphaea colorata* (*N. colorata*). Meanwhile, we also constructed unbalanced datasets for the following four species: *Glycine max* (*G. max*), *Malus domestica* (*M. domestica*), *Asparagus officinalis* (*A. officinalis*), and *Lupinus angustifolius* (*L. angustifolius*). In total, 949,115 sequences were downloaded (104,757 lncRNAs and 844,358 mRNAs) across the benchmark dataset, independent testing dataset, and unbalanced dataset. Since the sequence data may originate from the same transcriptome, removing duplicate sequences is essential. Therefore, we first employed CD-HIT (v4.8.1) software to eliminate redundant sequences, setting the similarity threshold to 0.9. Second, to ensure data quality, we eliminated sequences with low sequencing quality. Finally, to facilitate smooth data processing, we randomly selected sequences with lengths between 200 nt and 6000 nt from the dataset and matched lncRNA counts by randomly selecting mRNA sequences to balance the dataset. After eliminating redundant data, abnormal sequences (such as those with short lengths or low sequencing quality containing many unknown bases, indicated by N, X, or other symbols), and balancing the dataset, we obtained a benchmark dataset of 108,024 sequences, an independent dataset of 34,600 sequences, and an unbalanced dataset of 28,800 sequences. For more detailed information about the dataset, please refer to Supplementary Materials, Tables S1–S3. Table 1 provides details of the benchmark dataset for each species, Table 2 presents details of the independent test dataset, and Table 3 outlines the relevant details of the unbalanced dataset. All datasets are independent of each other, with no duplicate data present.

### 3.3. Multi-Feature Fusion

The RNA sequence feature extraction method plays a crucial role in subsequent prediction tasks. Currently, those methods can generally be divided into the following three categories:

1. Features based on biological properties. The features of open reading frames (ORFs) in RNA sequences are commonly used in computational biology to characterize them. ORFs refer to continuous nucleotide sequences between the start codon and the stop codon in an RNA sequence, and research has shown that these sequences have the potential to encode proteins. ORFs are contiguous nucleotide sequences between a start codon (e.g., AUG) and a stop codon (e.g., UAA) with potential to encode proteins. These features mainly include the length, coverage, and conservation of ORFs. mRNAs have specific ORFs, whereas lncRNAs lack such specific sequences. For example, mRNAs typically have longer ORF segments than lncRNAs. In this study, ORFs were predicted using the Biopython library, which identifies potential coding regions by detecting start and stop codons across all reading frames based on the nucleotide sequence based on the standard RNA codon table.
2. Sequences-based features. RNA sequences refer to linear structures composed of nucleotide sequences, also known as primary structures. These features include the composition of nucleotide sequences, sequence length, and codon preference. Studies have confirmed that lncRNAs and mRNAs differ in terms of sequence length, nucleotide sequences composition, codon preference, and coverage.
3. Structure-based features. Structure-based features refer to the secondary structure (SS) formed by base pairing and intermolecular hydrogen bonding based on the nucleotide sequence. It includes different features, such as hairpins, stem loops, pseudoknots [24] and minimum free energy (MFE). Different RNA molecules have distinct secondary structures determined by their length and nucleotide sequences.

Single feature extraction methods may not fully capture both local and global features of sequences. Fusing multiple features can capture more sequence information. However, it should be noted that the higher the feature dimension, the more computational resources and model training time are required, thereby affecting the performance of prediction models. In this study, we designed a simple yet effective feature that utilizes a multi feature fusion method; this method takes into account local and global features and sequential information. The features of RNA sequences were extracted from three different perspectives: biological properties-based features (e.g., ORF count and coverage), sequence-based features (e.g., GC content, Z-curve, AUGC ratio, and nucleotide composition), and structure-based features (e.g., base pairs and MFE).

1. ORF Count

The ORF count feature refers to the number of ORFs in an RNA molecule. ORFs are contiguous nucleotide molecule regions in an RNA sequence that have the potential to be translated into proteins. An ORF is typically defined by a start codon (usually AUG, which encodes methionine) and a stop codon (such as UAA, UAG, or UGA). Calculating the number of ORFs in RNA can be used to analyze the functionality and potential protein-coding capacity of RNA molecules. A higher number of ORFs may indicate that the RNA molecule has more potential protein-coding sequences, while a lower number of ORFs may suggest that the RNA primarily functions in a non-coding capacity.

2. ORF Coverage

The ORF coverage refers to the relative proportion of ORFs within a sequence and serves as a feature to measure the extent of potential coding regions. Sequences with high ORF coverage often contain more potential coding regions, while sequences with low coverage indicate fewer potential coding regions. Additionally, a high coverage rate implies a higher likelihood that the sequence is cRNA, whereas a lower coverage rate suggests a lower likelihood. The expression of ORF Coverage is given by Equation (1), as follows:

(1) $$ORF\;Coverage=\frac{L_{ORFs}}{L}\times 100\%$$

where $L_{ORFs}$ denotes the total length of ORFs and *L* denotes the length of the sequence.

3. ORF Length

The ORF length refers to the length of ORFs within a sequence and serves as a feature to measure the size of potential coding regions. Sequences with longer ORFs often indicate the presence of larger potential coding regions, which may correspond to functional genes or proteins, while sequences with shorter ORFs suggest smaller or less significant coding potential. Furthermore, a greater ORF length implies a higher likelihood that the sequence encodes a functional protein, while a shorter ORF length suggests a lower likelihood. The calculation of ORF length is determined by identifying the nucleotide distance from the start codon to the stop codon in a given reading frame.

4. Sequence Length

The length feature of an RNA sequence refers to the number of nucleotides (nt) in the RNA molecule, which plays an important role in distinguishing coding RNA from non-coding RNA. Studies have shown that mRNA is usually longer and has a specific length range used to encode proteins, while lncRNA has a wider length range and is involved in regulation and other biological functions. The length of the sequence can be expressed using the following Equation (2):

(2) Sequence Length=∑A+∑U+∑C+∑G

where, ∑A, ∑U, ∑C, and ∑G denote the sum of four bases.

5. GC Content

GC content refers to the percentage of G and C nucleotides in the nucleotide sequence, and RNA fragments from the same biological species often exhibit specific GC content. Research has shown that sequences with higher GC content tend to exhibit greater density and stability. The GC content can be expressed using the following Equation (3):

(3) $$GC\;Content=\frac{\sum G+\sum C}{\sum A+\sum U+\sum C+\sum G}$$

where, ∑A, ∑U, ∑C, and ∑G denote the sum of four bases.

6. Z-curve

The Z-curve [25] is an RNA sequence representation method based on the Z curve, which converts an RNA sequence into a three-dimensional curve in space. The Z-curve is a three-dimensional curve in space that exhibits rich folding structures and reflects the local details and general characteristics of the nucleotide distribution in an RNA sequence. It is defined by the following Equation (4):

(4) $$\left\{\begin{array}{c} x\;axis=\left(\sum A+\sum G\right)-\left(\sum C+\sum U\right)\\ y\;axis=\left(\sum A+\sum C\right)-\left(\sum G+\sum U\right)\\ z\;axis=\left(\sum A+\sum U\right)-\left(\sum G+\sum C\right) \end{array}\right.$$

where, ∑A, ∑U, ∑C, and ∑G denote the sum of four bases.

7. AUGC Ratio

The AUGC ratio refers to the proportional relationship between the sums of A and U quantities in a sequence and the sums of G and C quantities. The formula is defined by the following Equation (5):

(5) $$AUGC\;Ratio=\frac{\sum A+\sum U}{\sum G+\sum C}$$

where, ∑A, ∑U, ∑C, and ∑G denote the sum of four bases.

8. Nucleic Acid Composition

The nucleotide acid composition (NAC) method considers the frequency or count of individual nucleotides in a sequence. In an RNA sequence, there are four natural nucleotides: A, C, G, and U. Therefore, the NAC method can be expressed using the following Equation (6):

(6) $$f_{x}=\frac{N_{x}}{L}x\in \left\{A,C,G,U\right\}$$

where $N_{x}$ denotes the frequency of the nucleotide, and *L* denotes the length of an RNA sequence.

9. Di-nucleotide Composition

The di-nucleotide composition (DNC) method refers to the frequency of two adjacent nucleotides appearing in the sequence. There are sixteen possible combinations of four nucleotides, and the DNC method can be expressed using the following Equation (7):

(7) $$f_{xy}=\frac{N_{xy}}{L-2}\;x,y\in \left\{A,U,G,C\right\}$$

where $f_{xy}$ denotes the frequency of the nucleotide combination (x, y), $N_{xy}$ denotes the count of occurrences of the combination (x, y), and *L* denotes the length of an RNA sequence.

10. Tri-nucleotide Composition

The tri-nucleotide composition (TNC) method [26] is similar to DNC. This method refers to the frequency of three adjacent nucleotides appearing in the sequence, for example AAA, AAC, etc. Thus, there are sixty-four possible combinations of four nucleotides, and the TNC method can be expressed using the following Equation (8):

(8) $$f_{xyz}=\frac{N_{xyz}}{L-3}\;x,y,z\in \left\{A,U,G,C\right\}$$

where $f_{xyz}$ denotes the frequency of the nucleotide combination (x, y, z), $N_{xyz}$ denotes the count of occurrences of the combination (x, y, z), and *L* denotes the length of an RNA sequence.

11. Structure-based Features

In this study, structure-based features were generated using RNAFold [27] and represented in dot-bracket notation. RNAFold is a widely used RNA structure prediction tool that predicts RNA secondary structure and MFE by considering factors such as binding energy and sequence accessibility. We extracted the following six features from the generated secondary structure: the number of base pairs, the number of AU pairs, the number of GC pairs, the number of internal loops, the number of external loops, and the number of unpaired bases.

Additionally, we included MFE, which quantifies the minimum energy required to stabilize the most stable structure of RNA. To improve the convergence speed of the LMFE, we introduced a normalization method by calculating the ratio of MFE to sequence length [28,29]. The normalization ensures comparability across RNA sequences, and the normalized MFE can be defined by the following Equation (9):

(9) $$G_{NORM}=\frac{G_{MFE}}{L}$$

where $G_{NORM}$ is the normalized minimum free energy, $G_{MFE}$ is the non-normalized minimum free energy, and *L* represents the length of the sequence. Table 4 shows the feature extraction methods and the number of features used in this study.

### 3.4. Ensemble Learning Method

Ensemble learning is a method that combines multiple weak predictors into a strong predictor, and decision trees (DT) can be chosen as one of the weak predictors. This method integrates data fusion, modeling, and mining into a framework. The method extracts a set of features using various feature extraction methods and generates weak predictors using multiple learning methods. Finally, a strong predictor with enhanced predictive ability is generated by iteratively combining the prediction results of multiple weak predictors using such methods as adaptive fusion or voting [30].

Currently, commonly used ensemble learning methods include bagging, boosting, and stacking, each with its own characteristics [31,32].

The bagging method obtains multiple training subsets through resampling, with each subset being independently trained, reducing the variance of the base predictor and improving generalization error. Additionally, weak predictors can be run in parallel. The bagging method first generates multiple weak predictors, and a strong predictor is created by aggregating the predictions of these weak predictors through voting or averaging. This approach improves overall performance by reducing the variance of the predictors.

The boosting method iteratively trains multiple weak classifiers while keeping the training set constant throughout the iterations. The sample weights are adjusted based on the results of the previous iteration, focusing on the misclassified samples from the prior learning process and assigning them higher weights. In each iteration, the misclassified samples from the previous round are relearned by the newly generated predictor. This iterative process enhances the predictive performance of weak predictors and ultimately leads to a strong predictor. Additionally, the overall performance is enhanced by reducing the bias of the predictors.

The stacking method combines multiple weak predictors of varying types into a strong predictor. During the training process, a new training set is generated and utilized to train weak predictors. Stacking is a multi-layered learning method that uses the outputs of multiple well-performing weak predictors, including the predicted probabilities of labels, as inputs for the next-layer model. This approach effectively enhances the performance of the prediction method.

In this study, we employed XGBoost as the prediction method, a specific implementation of the boosting method. Similar to other ensemble learning methods, XGBoost iteratively trains multiple weak classifiers and ultimately obtains a strong classifier. However, compared to gradient boosting decision tree (GBDT) methods, XGBoost offers advantages in terms of its model structure, loss function optimization, and regularization strategies. Regarding model structure, XGBoost utilizes GBDT [33] as the base classifier rather than integrating multiple weighted weak classifiers. During the iteration process, new weak classifiers are gradually constructed by optimizing the gradient of the loss function rather than generating weak classifiers through weighting or averaging. Regarding loss function, XGBoost adopts second-order Taylor expansion for the loss function and optimizes the loss function using both first-order and second-order derivatives.

Additionally, XGBoost incorporates L1 and L2 regularization strategies. On one hand, model complexity is constrained through regularization terms to prevent overfitting and improve the model’s generalization ability. On the other hand, during the iteration process, the contribution of weak classifiers is limited by controlling the learning rate (lr), thereby enhancing the robustness. Taking binary classification as an example, the model’s working principle is as follows. Let us assume that we have an RNA dataset X with *N* samples, as shown in the following Equation (10):

(10) $$\left(x_{1},y_{1}\right),\left(x_{2},y_{2}\right),\left(x_{3},y_{3}\right),\cdots ,\left(x_{i},y_{i}\right),\cdots ,\left(x_{N},y_{N}\right)\;y_{i}\in \left\{0,1\right\}$$

1. At the initial stage, the *lr*, maximum tree depth (max_depth), number of estimators (n_estimators), and other parameters are initialized. The initial predicted value *F*_0_ (*X*) = 0.
2. Perform iterations for the current generated tree model t:
  - Compute the gradients $g_{t}(x_{i})$ and second derivatives $h_{t}(x_{i}$) of the current model.
  - Fit a tree model using the training dataset (X, $g_{t}$, $h_{t}$) with a maximum depth constraint of max_depth.
  - Use the tree model to make predictions on the training samples. For a sample $x_{i}$, its predicted value is denoted as $f_{t}(x_{i})$.
  - Update the predicted values of the model. For a sample $x_{i}$, the updated predicted value is given by $F_{t}(x_{i})=F_{t-1}(x_{i})+lr\times f_{t}(x_{i})$.
  - Update the approximation of the loss function to minimize it. The loss function is defined by the following Equation (11):

(11) $${Obj}_{t}=\sum_{i=1}^{N}L\left(y_{i},\;F_{t}\left(x_{i}\right)\right)+\Omega {(f}_{t})$$

where $\sum_{i=1}^{N}L\left(y_{i},\;F_{t}\left(x_{i}\right)\right)$ denotes the overall loss and $\Omega (f_{t})$ is the regularization term for the t-th tree model, which measures the complexity of the generated tree $f_{t}$ during the current iteration. $\Omega (f_{t})$ can be defined as in the following Equation (12):

(12) $$\Omega {(f}_{t})=\gamma T+\frac{1}{2}\lambda \sum_{i}^{T}\omega_{j}^{2}$$

where T is the number of leaves in the t-th tree, $\omega_{t}$ is the score of the j-th leaf, and γ and λ are regularization hyperparameters.

3. Check if the maximum number of iterations has been reached. If it has, stop the iteration; otherwise, continue the iteration.
4. After the iterations have been completed, a strong predictor is obtained.

### 3.5. Evaluation Metrics

To comprehensively evaluate the performance and effectiveness of LMFE, we employed commonly used evaluation metrics to assess its predictive performance. These evaluation metrics include the ACC, sensitivity (SN), specificity (SP), F1_score_, matthews correlation coefficient (MCC), and ROC curve. When plotting the ROC curve and calculating the area under the curve (AUC), it is necessary to calculate the true positive rate (TPR) and false positive rate (FPR) of the training samples. The TPR represents the proportion of samples correctly predicted as positive, while the FPR represents the proportion of negative samples incorrectly predicted as positive. The definitions of the evaluation metrics are given by the following Equations (13)–(21):

(13) $$ACC=\frac{TP+TN}{TP+FN+FP+FN}\times 100\%$$

(14) $$Precision=\frac{TP}{TP+FN}\times 100\%$$

(15) $$SN=\frac{TP}{TP+FN}\times 100\%$$

(16) $$SP=\frac{TN}{TN+FP}\times 100\%$$

(17) $${F1}_{score}=\frac{2\times TP}{2\times TP+FP+FN}$$

(18) $$Recall=\frac{TP}{TP+FN}$$

(19) $$MCC=\frac{TP+TN+FP+FN}{\sqrt{\left(TP+FP\right)\times \left(TN+FN\right)\times \left(TP+FN\right)\times \left(TN+FP\right)}}$$

(20) $$TPR=\frac{TP}{FP+FN}\times 100\%$$

(21) $$FPR=\frac{FP}{FP+TN}\times 100\%$$

where TP represents the number of samples that are actually positive and that are correctly predicted as positive. TN represents the number of samples that are actually negative and that are correctly predicted as negative. FN represents the number of samples that are actually negative but that are incorrectly predicted as positive. FP represents the number of samples that are actually positive but that are incorrectly predicted as negative.

## 4. Results and Analysis

In this section, we elaborate on the experimental settings and analyze the experimental results. This study used Python 3.11 and PyCharm 2024.1 as experimental environment. The computer operating system was a Windows Server 2019, equipped with an Intel (R) Xeon (R) Silver 4114 @ 2.20 GHz CPU and 32 GB of RAM. The experimental design is divided into five phases, as follows.

In the initial phase, we conducted lncRNA sequence prediction experiments using the benchmark dataset to assess the performance of the XGBoost method and compared it with other machine learning methods. Various methods, such as SVM and adaptive gradient boosting (AB) have been widely utilized in various fields of bioinformatics research in recent years, particularly in lncRNA prediction, due to their advantages of rapid training, low data dependency, and strong interpretability. However, reliance on shallow machine learning methods alone is susceptible to data quality issues and may lead to overfitting. In contrast, ensemble learning methods can overcome the limitations of shallow machine learning methods while retaining their advantages, such as rapid training, low data dependency, and strong interpretability. To comprehensively understand the predictive performance of LMFE, this study compared it with four shallow machine learning methods, namely K-nearest neighbor (KNN), decision tree (DT), Gaussian naive Bayes (NB), and SVM. Additionally, four ensemble learning methods were employed, namely random forest (RF) [34], bagging (BG), AdaBoost (AB), and gradient boosting decision trees (GBDTs). This comparative analysis was conducted to verify the effectiveness and superiority of the XGBoost method. After choosing the prediction methods and constructing the framework, we further analyzed the importance of the selected features.

In the second phase, we focused on analyzing the correlation among features and the impact of redundant features on LMFE performance. We examined the original 100-feature collection and identified highly correlated features (such as GC content, Z-curve, and the AUGC ratio) using the Pearson correlation coefficient. A reduced dataset was generated by filtering out redundant features with correlation coefficients greater than 0.8, followed by the gradual reintroduction of these features to evaluate their respective contributions. This stage aims to elucidate the balance between redundancy and predictive ability.

In the third phase, to evaluate LMFE’s ability to identify lncRNAs across species, we separately trained LMFE using data from each of the ten different species in the benchmark dataset and conducted cross-species validation on datasets of other species individually [35].

In the fourth phase, due to challenges in accurately reflecting the model’s performance in practical applications using a balanced dataset, this part of the experiment focused on constructing unbalanced datasets. This was performed to verify the model’s performance on unbalanced datasets, particularly to evaluate its generalization ability and capacity to identify minority classes.

In the final part of the experiment, we compared the performance of LMFE with state-of-the-art prediction methods. We selected commonly used methods, including CPC2, PLEKv2, LGC, CNCI, and PlncRNA-HDeep.

### 4.1. Analysis of Performance of Ensemble Learning Method

In the first part of the experiment, we trained and validated LMFE performance using a benchmark dataset. To optimize XGBoost parameters and prevent overfitting, we employed random parameter search [36] with 5-fold cross-validation to adjust key parameters, while keeping the remaining parameters at their default values. The explored parameter ranges were as follows:

- n_estimators (Number of base learners): 100, 200, 300, 500.
- max_depth (Tree depth): 3, 4, 5, 6, 7, 8, 9.
- learning_rate (Learning rate): 0.1, 0.01, 0.001.
- subsample (Subsample ratio): 0.5, 0.6, 0.7, 0.8, 0.9, 1.0.
- colsample_bytree (Column sampling ratio): 0.5, 0.6, 0.7, 0.8, 0.9, 1.0.
- alpha (L1 regularization parameter): 0, 0.1, 1, 10.
- lambda (L2 regularization parameter): 0.1, 1, 10.

Through this process, we identified the following optimal parameter combination: n_estimators = 300, max_depth = 8, learning_rate = 0.1, subsample = 0.7, colsample_bytree = 0.8, alpha = 0, and lambda = 1. This configuration ensured robust performance evaluation and mitigated overfitting, achieving an average ACC of 99.42% on the benchmark dataset, as shown in Table 5.

To compare the impact of different machine learning methods on LMFE, this study analyzed the performance of the XGBoost against four shallow machine learning methods and four ensemble learning methods under 5-fold cross-validation. The evaluation metrics SN, SP, ACC, F1_score_, and MCC were used to assess the predictive capability of the proposed method. To ensure the reliability and consistency, default parameters were preserved for all comparison methods utilized. The comparison results are shown in Table 6.

**Figure 2:**
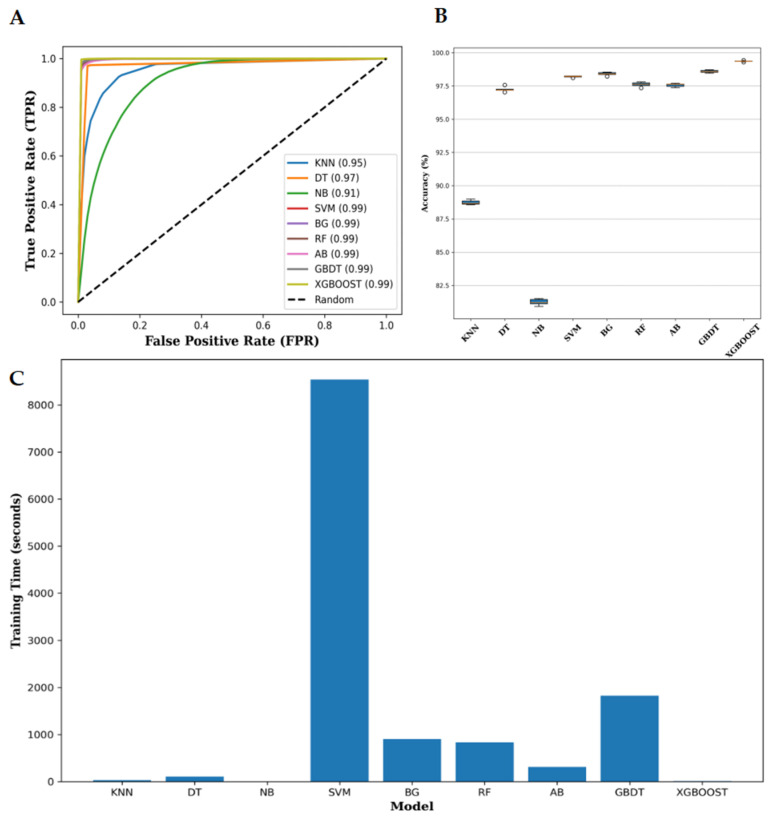
The comparison results between XGBoost and other methods. (**A**) The comparison of the ROC curves and AUC values of XGBoost and other methods. (**B**) The ACC compared with other mainstream methods; it can be observed that XGBoost achieves good performance, while GBDT, BG, and SVM are slightly better than other methods. (**C**) The time consumed by different classifiers on the training set. From the figure, it can be seen that SVM takes the longest time, while XGBoost’s advantage lies in its efficient performance.

Furthermore, this study also verified the ROC curve and box plot of XGBoost’s ACC compared with other mainstream methods, as well as the average time comparison chart for each prediction method. The results are presented in Figure 2.

Overall, based on the comparison results, XGBoost demonstrates outstanding performance in terms of ROC curves and AUC values, achieving an AUC of 0.99, which indicates excellent prediction capability. SVM and other ensemble learning methods also demonstrate strong performance, while KNN, DT, and NB have lower AUC values (ranging 0.91–0.95). In terms of ACC, XGBoost also performs exceptionally well, matching the ACC of GBDT, which is slightly better than that of other mainstream methods. The training time analysis shows that SVM takes the longest to train, which can be a disadvantage for scenarios requiring quick model updates, while XGBoost has a significant advantage in training efficiency, making it suitable for large datasets and real-time applications. Other classifiers, such as DT and RF, have training times that fall between those of XGBoost and SVM, demonstrating a balance between performance and efficiency.

In addition to evaluating the impact of different machine learning methods, this study conducted a comparative analysis of the performance under different feature combination. The benchmark dataset was divided into a training set and a testing set (7:3); LMFE was trained on the training set, and validation was conducted on the testing set.

**Figure 3:**
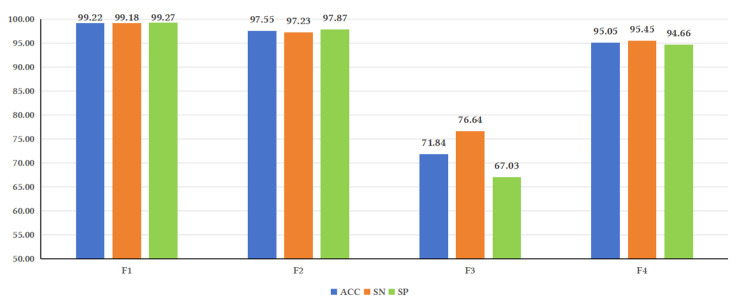
Illustrates the performance of the model under different feature fusions. Analysis of the evaluation metrics reveals that some metrics, such as ACC, SN, and SP, showed higher values for F1 compared to F2, F3, and F4. F2 yielded higher values than F3 and F4. These results indicate that sequence-based features have a significant impact on model performance. This could be attributed to studies that have observed the lack of secondary structure conservation in lncRNAs of certain species [37], suggesting that secondary structure may not be as important for predicting lncRNAs as previously believed. These findings are consistent with the results of this study.

The features combining biological properties, sequences, and secondary structures were collectively referred to as F1. The feature consisting of only sequence information was referred to as F2, the feature consisting of only secondary structures was referred to as F3, and the feature consisting of only biological properties was referred to as F4, as shown in Figure 3.

**Figure 4:**
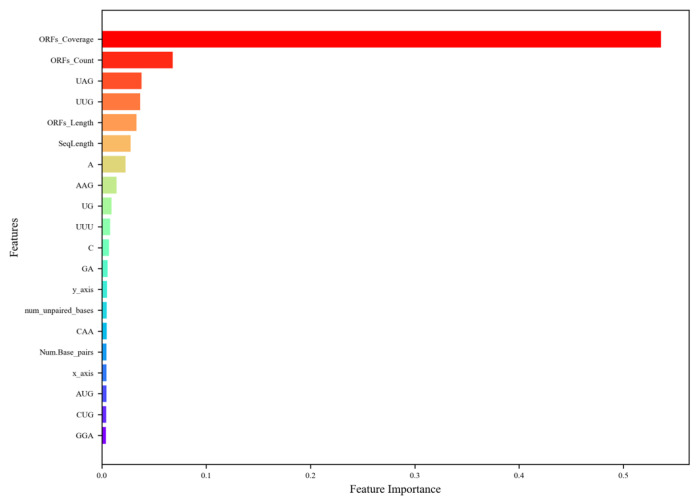
The ranking of the top 20 most important features. The descriptions of the feature abbreviations are as follows: ORFs_Coverage (ORF coverage), ORFs_Count (ORF count), ORFs_Length (ORF length), SeqLength (sequence length), y_axis (y asix), Num_unpaired_bases (the number of unpaired bases), Num_Base_pairs (the number of base pairs), and x_axis, (x axis). The figure highlights that ORF-related features dominate, with ORF coverage being the most significant, followed by ORF count and ORF length, reflecting their critical role in distinguishing lncRNAs from mRNAs due to the former’s lower ORF presence. Tri-nucleotide compositions, such as UAG and UUG, also rank highly, indicating their relevance in capturing sequence-level differences, particularly since stop codons, such as UAG, are more common in mRNAs. Sequence length and nucleotide compositions (e.g., A, C, GA) contribute moderately, while structural features (e.g., num_unpaired_bases, Num_Base_pairs) and Z-curve components (y_axis, x_axis) have lower importance (~0.03–0.04), suggesting that sequence-based features are more discriminatory than structural ones for lncRNA prediction in plants.

To further evaluate the importance of features, we ranked the top 20 most important features using XGBoost’s feature importance score, which is based on their frequency (weight) in the decision tree. As shown in Figure 4.

### 4.2. Analysis of Performance on Feature Redundancy

To systematically evaluate the influence of potentially redundant features, such as GC content, Z-curve, and AUCG ratio, when individual A, U, G, and C contents are provided, we conducted an experiment focused on feature correlation and its effect on the predictive performance of LMFE. The experiment began with an analysis of the original 100-dimensional feature set. We calculated the Pearson correlation coefficient (ρ) for all pairs of features to measure their linear relationships, as shown in the following Equation (22):

(22) $$\rho =\frac{cov(X,Y)}{\sigma X\sigma Y}$$

where cov(X,Y) represents covariance and σX and σY are standard deviations. Features exhibiting a correlation coefficient greater than 0.8 in absolute value were deemed highly correlated, and for each such pair, features exhibiting a correlation coefficient greater than 0.8 in absolute value were deemed highly correlated. For each of these pairs, to ensure the reliability, we randomly retained one of the features and kept the other one as a redundant feature. After filtering features, we retained a reduced dataset with 64 low-correlation features (denoted as $D_{reduced}$); the remaining 36 features were considered high correlation. We then trained and evaluated LMFE on $D_{reduced}$ and achieved an ACC of 96.30% and a precision of 95.45%.

**Figure 5:**
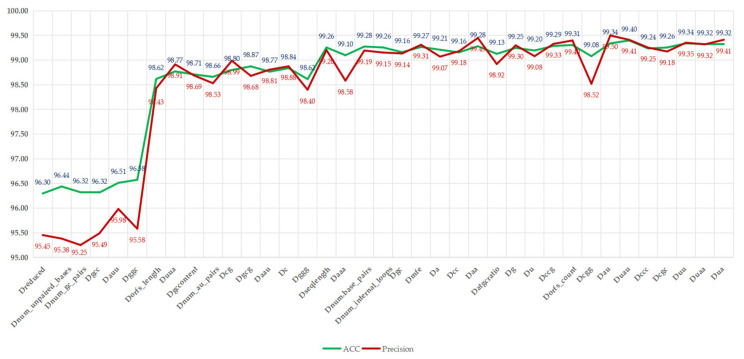
Illustrates that, with the reintroduction of redundant features, the ACC and precision metrics show an overall upward trend, increasing from 96.30% and 95.45% to 99.41% and 99.32%, respectively, with improvement rates of 3.11% and 3.87%. However, slight fluctuations were observed; for instance, adding “num_unpaired_bases” increased the ACC to 96.44%, while precision slightly decreased to 95.38%, indicating potential fluctuations due to its direct correlation with RNA structural stability. The most significant improvement occurred after the addition of “Dorfs_length”, with the ACC increasing from 95.58% to 98.43%, reflecting the crucial role of ORF-related features in distinguishing lncRNAs from mRNAs. However, with the addition of feature “C”, slight fluctuations in ACC and precision were noted, decreasing from 98.84% and 98.88% to 98.62% and 98.40%, respectively. This indicates that these features may introduce noise or overfitting to certain samples, possibly due to their high correlation with existing features, such as nucleotide composition. As the reintroduction process neared its conclusion, with the introduction of features, such as “Dgcc” and “Duaa”, both ACC and precision were restored. The ACC ultimately stabilized at 99.32% to 99.40%, with precision stabilizing at around 99.32% to 99.41%, nearing the performance of all features, which had 99.32% ACC and 99.41% precision. This analysis indicates that, although some redundant features (such as “Dnum_au_pairs”) temporarily compromise precision, the overall trend supports their inclusion in the complete feature set, as they collectively enhance LMFE’s ability to capture subtle patterns in RNA sequences, especially when balanced with biologically significant features, such as ORF coverage. These findings emphasize the robustness of XGBoost in processing relevant features and suggest that careful feature selection can alleviate transient performance degradation. We will further explore this consideration in future work by integrating advanced feature selection techniques.

Subsequently, we reintroduced the filtered redundant features into the Dreduced dataset one by one, thereby creating a series of datasets (e.g., Dnum_unpaired_bases, which indicates that num_unpaired_bases features are added to the Dreduced dataset. Dnum_gc_pairs indicates that num_gc_pairs features are added after num_unpaired_bases features, and so forth). For each accumulation of redundant features, the LMFE was retrained and evaluated using the same methodology. This process enabled us to quantify the incremental impact of each redundant feature on LMFE performance. The experimental results are shown in Figure 5; for more detailed information about other evaluation metrics, please refer to Table S4 in the Supplementary Materials.

### 4.3. Analysis of Performance on Cross-Species Dataset

To verify the generalization ability of LMFE, we conducted experiments using data from 10 different plants in a benchmark dataset to validate its cross-species generalization ability.

Specifically, we first trained LMFE on datasets from ten different plant species and then used these trained models to validate their cross-species generalization ability on other plant species. We used ACC as the evaluation metric, and the performance of LMFE on various evaluation metrics on the benchmark dataset is shown in Table 7.

**Figure 6:**
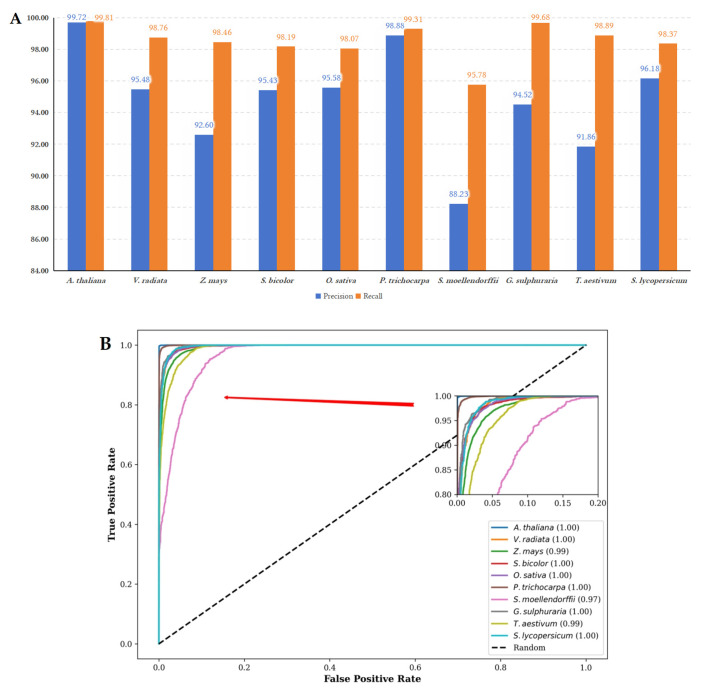
(**A**) The performance metrics of precision and recall for LMFE trained on the *A. thaliana* dataset and evaluated on other species. The LMFE demonstrates excellent performance on the *A. thaliana* dataset, with precision and recall values approaching 99.72% and 99.81%, respectively. This indicates a strong adaptability to the characteristics of the species. The verification results for other species reveal that the precision remains relatively stable, with a slight decrease observed in *S. lycopersicum*. The highest precision is recorded at 99.31% for *P. trichocarpa*, while the lowest precision is 88.23% for *S. mollendorffii*. In terms of recall, all species maintain values above 98%, with the highest recall at 99.68% for *G. sulphuraria* and the lowest recall at 95.78% for *S. mollendorffii*. Overall, LMFE exhibits a consistent trend in precision and recall across different species, with only minor fluctuations, suggesting its robustness and potential for broad applicability in various biological contexts. (**B**) The ROC curves for LMFE trained on the *A. thaliana* dataset and assessed across various species. The ROC curve serves as a graphical representation of performance, plotting the true positive rate against the false positive rate at various threshold settings. The ROC curve for *A. thaliana* is nearly perfect, signifying that LMFE excels at distinguishing positive samples with minimal false positives. The AUC value of 1.00 indicates that LMFE can correctly identify the majority of positive samples. Other species, such as *V. radiata* and *Z. mays*, also demonstrate strong performances, with AUC values of 1.00 and 0.99, respectively. This suggests that LMFE maintains a high level of accuracy for these species. However, the ROC curve for *S. moellendorffii* is comparatively lower, with an AUC value of 0.97. While still indicating good performance, this suggests that LMFE’s performance on this species is slightly less robust than on others, potentially due to differences in data characteristics. Overall, LMFE exhibits excellent training results on the *A. thaliana* dataset and demonstrates strong performance across different species.

To validate the performance on different datasets, we further analyzed the ROC curves, precision, and recall on datasets from other species, as shown in Figure 6.

Overall, Figure 6 demonstrates that LMFE has satisfactory performance in cross-species validation, indicating that LMFE has a certain ability to predict lncRNAs across species. The experimental results of cross-species validation among other species are presented in Figures S1–S9 in the Supplementary Materials.

### 4.4. Analysis of Performance on Unbalanced Dataset

In the fourth stage of the experiment, since XGBoost is essentially a tree-based model, in classification problems, when the number of samples contained in a certain category in the dataset is significantly less than that of other categories, the accuracy of this method will drop significantly. Therefore, this study assessed the predictive capability of LMFE on an unbalanced dataset. To address the issue of unbalanced datasets, we employed the synthetic minority oversampling technique (SMOTE) as a data resampling method. SMOTE, introduced by Nitesh V. Chawla et al. [38], is a method designed specifically for addressing imbalanced datasets. The primary objective is to augment the number of minority class samples by generating synthetic instances, thereby balancing the class distribution. SMOTE generates new minority class instances by interpolating within the feature space, rather than merely duplicating existing minority class samples. SMOTE has been extensively utilized in computational biology and data mining, particularly for addressing unbalanced data challenges. By generating synthetic instances, SMOTE enables LMFE to more effectively learn the characteristics of the minority class, thereby enhancing performance. We utilized the unbalanced datasets described in Table 3 as test datasets and applied our proposed method to predict the class labels within these datasets. In order to effectively compare the impact of SMOTE technology on improving performance, we compared the performance before and after the introduction of SMOTE technology. The two groups of experiments were named EXP NO-SMOTE, which was tested on unbalanced datasets without SMOTE, and EXP WITH-SMOTE, where SMOTE was employed to balance the datasets prior to testing. We used ACC, recall, and F1_score_ as evaluation metrics to measure performance. The results of these experiments are presented in Table 8.

**Figure 7:**
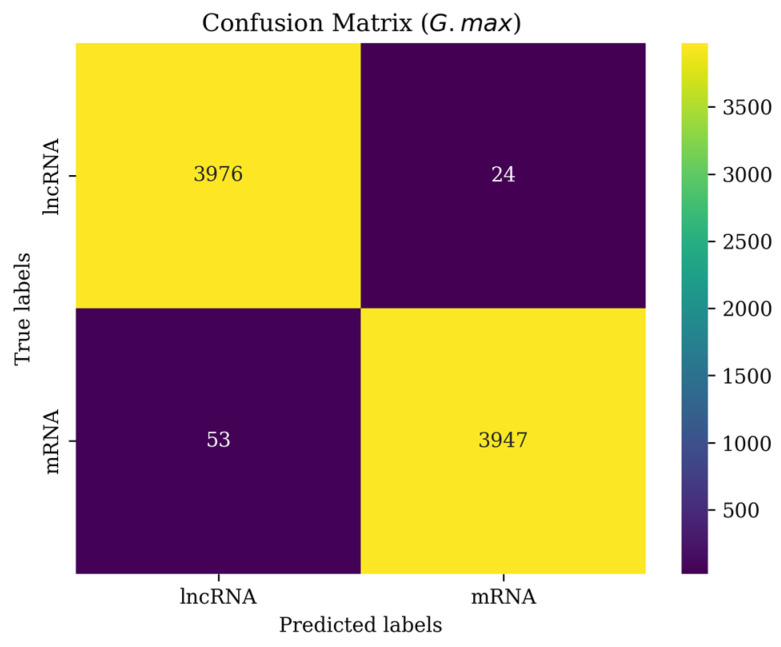
The confusion matrix for LMFE’s performance on *G. max* dataset after applying SMOTE, illustrating its ability to classify lncRNA and mRNA sequences. After SMOTE, the dataset was balanced to include 4000 true lncRNA samples and 4000 true mRNA samples. The matrix shows that out of 4000 true lncRNA samples, 3976 were correctly predicted as lncRNA (true positives), while 24 were misclassified as mRNA (false negatives). Conversely, out of 4000 true mRNA samples, 3947 were correctly predicted as mRNA (true negatives), but 53 were misclassified as lncRNA (false positives). The high values along the diagonal (3976 and 3947) and the low off-diagonal values (24 and 53) indicate a low error rate, demonstrating that LMFE accurately distinguishes between lncRNA and mRNA in most cases, with strong recognition ability for both categories despite the unbalanced dataset after applying SMOTE. Experimental results for other species are shown in Figures S10–S12 in the Supplementary Materials.

To further demonstrate the performance of LMFE on the unbalanced dataset, this study provides a confusion matrix to illustrate the performance of LMFE after applying SMOTE. The result is shown in Figure 7.

### 4.5. Compare with State-of-the-Art Method

To compare the prediction accuracy between LMFE and commonly used state-of-the-art methods for plant lncRNA prediction, this study selected CPC2, PLEKv2, LGC, CNCI, and PlncRNA-HDeep as benchmark tools due to their widespread application in this domain. CPC2 offers a user-friendly web server and a standalone Python package for lncRNA prediction. PLEKv2 is available as a command-line tool implemented in Python, requiring specific dependencies for lncRNA prediction, as demonstrated in this study. LGC provides a web-based interface for submitting FASTA sequences, making it accessible for plant genome analysis. CNCI is available as a standalone software package written in Python, supporting efficient lncRNA prediction across species. PlncRNA-Hdeep can be run locally via command-line scripts after preparing input FASTA files and corresponding labels. The detailed parameters and usage instructions for these tools are provided in Supplementary Materials Table S5. The comparison was performed using an independent dataset, with ACC as the evaluation metric. The performance is shown in Table 9.

**Figure 8:**
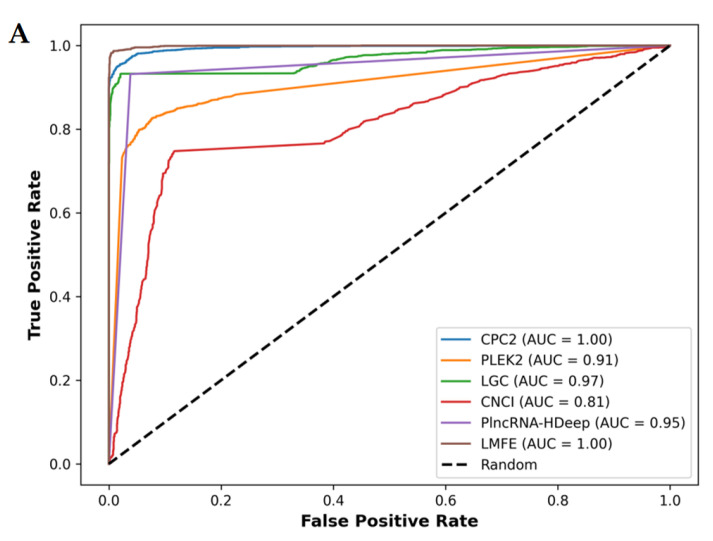

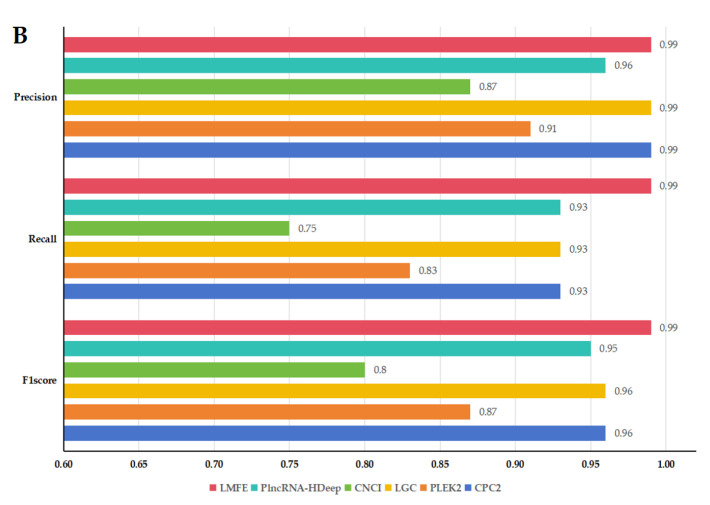
(**A**) Demonstrates that in the *V. angularis*, the AUC value of LMFE is 1.00, indicating excellent performance in distinguishing positive and negative samples, achieving perfect true positive rates at nearly all thresholds. CPC2, LGC, and PlncRNA-Hdeep also show perfect capabilities. In contrast, CNCI obtained a slightly lower AUC value of 0.87. (**B**) Confirms that LMFE demonstrated superior performance across all metrics, achieving a score of 0.99, reflecting extremely high accuracy and comprehensiveness. LGC and CPC2 closely followed, with a precision of 0.99 and recall and F1_score_s of 0.93 and 0.96, respectively, indicating a good balance. PlncRNA-Hdeep achieved a precision of 0.96, recall of 0.93, and F1_score_ of 0.95, showcasing its effectiveness. PLEKv2 achieved a precision of 0.91, recall of 0.83, and F1_score_ of 0.87; its low recall suggests that its ability to identify positive samples requires optimization. Conversely, CNCI exhibited the poorest performance, with a precision of 0.87, recall of only 0.75, and F1_score_ of 0.80, indicating significant deficiencies in identifying positive samples.

We further provide the ROC curve and the comparison of recall and F1_score_ values as shown in Figure 8.

For comparisons results on the other five datasets, we have provided Figures S13–S17 in the Supplementary Materials.

## 5. Conclusions and Discussion

This study introduces LMFE, a novel method for predicting plant lncRNAs using multi-feature fusion and ensemble learning. LMFE achieves high ACC (99.42% on the benchmark dataset, Table 5) and robust cross-species performance (89.30% to 99.81%, Table 7), outperforming state-of-the-art methods, such as CPC2 and PLEKv2 (Table 9). Its feature extraction integrates biological properties-based, sequence-based, and structure-based features with XGBoost, ensuring superior performance (Table 6). Notably, redundant features minimally impact LMFE’s performance (Figure 5 and Supplementary Materials Table S4), as the model prioritizes discriminative features, such as ORF coverage. This robustness is beneficial in cross-species validation, where redundancy helps to capture diverse sequence patterns, maintaining high performance despite variations.

While deep learning offers automatic feature learning [20,39], its challenges—long training times, high data needs, and limited interpretability—make ensemble learning a practical choice for plant lncRNA prediction, given the field’s early-stage data constraints. To enhance LMFE, integrating ensemble deep learning models (e.g., CNNs, RNNs) could improve cross-species generalization, while larger datasets, including species, such as *Ruta graveolens* L. [40], may enrich feature diversity. Interdisciplinary insights, such as phenotype progression [41], can further link predictions to plant traits, advancing lncRNA research and applications.

## Figures and Tables

**Table 1:** Benchmark dataset.

Species	Dataset		Total
	Positive Data	Negative Data	
*A. thaliana*	6775	6775	13,550
*V. radiata*	4600	4600	9200
*Z. mays*	11,572	11,572	23,144
*S. bicolor*	5400	5400	10,800
*O. sativa*	6003	6003	12,006
*P. trichocarpa*	5615	5615	11,230
*S. moellendorffii*	2300	2300	4600
*G. sulphuraria*	1870	1870	3740
*T. aestivum*	6500	6500	13,000
*S. lycopersicum*	3377	3377	6754

Table 1 presents the data obtained from public datasets in this study. After rigorous data preprocessing and balancing, we obtained a dataset of ten species for training and evaluating LMFE performance.

**Table 2:** Independent testing dataset.

Species	Dataset		Total
	Positive Data	Negative Data	
*V. angularis*	2000	2000	4000
*S. indicum*	3400	3400	6800
*B. distachyon*	3000	3000	6000
*M. acuminata*	5600	5600	11,200
*M. polymorpha*	1200	1200	2400
*N. colorata*	2100	2100	4200

Table 2 presents the independent test dataset used to evaluate LMFE performance, with all data processed using the same method as in the benchmark dataset.

**Table 3:** Unbalanced dataset.

Species	Dataset		Total
	Positive Data	Negative Data	
*G. max*	4000	2000	6000
*M. domestica*	2000	5500	7500
*A. officinalis*	6500	2300	8800
*L. angustifolius*	1700	4800	6500

Table 3 presents the composition of the unbalanced dataset. To verify the performance of the method in the unbalanced dataset, we constructed datasets from two perspectives: an unbalanced positive dataset and an unbalanced negative dataset.

**Table 4:** Feature extraction methods.

Classification	Method	Number of Features
Biological properties-based method	ORF count	1
	ORF coverage	1
	ORF length	1
Sequence-based method	Sequence length	1
	GC content	1
	Z-curve	3
	AUGC ratio	1
	NAC	4
	DNC	16
	TNC	64
Structure-based method	SS	6
	MFE	1
Total		100

Table 4 presents the features and their classifications introduced in this article, which we classify into 3 main categories, namely the biological properties-based method (3 features), sequence-based method (87 features), and structure-based method (10 features). The total number of features is 100.

**Table 5:** Performance of LMFE on the benchmark dataset.

Dataset	ACC (%)	SN (%)	SP (%)	F1_score_	MCC
Benchmark dataset	99.42	99.36	99.48	0.99	0.98

Table 5 illustrates that LMFE achieved an ACC of 99.42% on the benchmark, indicating excellent performance overall, and that satisfactory results were obtained for all evaluation metrics.

**Table 6:** Comparison Between XGBoost and other methods.

Method	ACC (%)	SN (%)	SP (%)	F1_score_	MCC
KNN	88.74	85.08	92.40	0.88	0.78
DT	97.24	97.27	97.22	0.97	0.94
NB	81.24	76.63	85.85	0.80	0.63
SVM	98.20	99.00	97.40	0.98	0.96
BG	98.41	98.14	98.68	0.98	0.97
RF	97.61	98.72	96.50	0.98	0.95
AB	97.56	97.96	97.16	0.98	0.95
GBDT	98.59	98.74	98.44	0.99	0.97
XGBoost	**99.3** **6**	**99.3** **1**	**99.4** **1**	**0.99**	**0.99**

Table 6 presents a performance comparison of XGBoost and other mainstream methods on the benchmark dataset. As indicated in the table, the average values of XGBoost across various evaluation metrics are slightly higher than those of the other methods. Specifically, compared to GBDT, the ACC is 0.77% higher, and the SN is 0.57% higher. The BG, RF, and AB demonstrated good performance. Additionally, SVM, one of the commonly used methods for lncRNA classification, has also exhibited good performance. The method exhibiting the lowest performance is NB. In terms of ACC, XGBoost performs 18.12% better than the NB. The values corresponding to the highest evaluation metrics in the table are highlighted in bold for emphasis.

**Table 7:** The performance of LMFE on the benchmark dataset.

Training Species	Metrics	Testing Species									
		*A. thaliana*	*V. radiata*	*Z. mays*	*S. bicolor*	*O. sativa*	*P. trichocarpa*	*S. moellendorffii*	*G. sulphuraria*	*T. aestivum*	*S. lycopersicum*
*A. thaliana*	ACC (%)	**99.76**	97.04	95.30	96.74	96.77	99.09	91.50	96.95	95.06	97.23
	SN (%)	**99.81**	98.76	98.46	98.19	98.07	99.31	95.78	99.68	98.89	98.37
	SP (%)	**99.72**	95.33	92.13	95.30	95.48	98.88	87.22	94.23	91.23	96.09
	F1_score_	**1.00**	0.97	0.95	0.97	0.97	0.99	0.92	0.97	0.95	0.97
	MCC	**1.00**	0.94	0.91	0.94	0.94	0.98	0.83	0.94	0.90	0.95
*V. radiata*	ACC (%)	98.12	**99.57**	94.96	95.08	95.74	98.46	92.87	97.19	94.98	97.65
	SN (%)	99.01	**99.67**	98.79	98.52	98.58	99.29	96.09	99.63	99.09	98.67
	SP (%)	97.23	**99.46**	91.13	91.65	92.90	97.63	89.65	94.76	90.86	96.62
	F1_score_	0.98	**1.00**	0.95	0.95	0.96	0.99	0.93	0.97	0.95	0.98
	MCC	0.96	**0.99**	0.90	0.90	0.92	0.97	0.86	0.95	0.90	0.95
*Z. mays*	ACC (%)	98.89	97.58	**99.61**	97.90	98.09	98.84	95.61	97.06	95.04	98.28
	SN (%)	98.51	98.09	**99.59**	97.96	98.78	98.38	96.22	99.14	98.08	98.05
	SP (%)	99.26	97.07	**99.62**	97.83	97.39	99.31	95.00	94.97	92.00	98.52
	F1_score_	0.99	0.98	**1.00**	0.98	0.98	0.99	0.96	0.97	0.95	0.98
	MCC	0.98	0.95	**0.99**	0.96	0.96	0.98	0.91	0.94	0.90	0.97
*S. bicolor*	ACC (%)	98.75	97.74	96.51	**99.66**	98.54	98.83	93.09	97.70	96.04	97.59
	SN (%)	98.86	98.41	98.36	**99.72**	99.00	98.75	96.65	99.47	98.63	97.99
	SP (%)	98.64	97.07	94.67	**99.59**	98.07	98.91	89.52	95.94	93.45	97.19
	F1_score_	0.99	0.98	0.97	**1.00**	0.99	0.99	0.93	0.98	0.96	0.98
	MCC	0.98	0.96	0.93	**0.99**	0.97	0.98	0.86	0.96	0.92	0.95
*O. sativa*	ACC (%)	98.60	98.14	96.16	97.72	**99.78**	98.49	91.98	97.62	96.15	97.57
	SN (%)	98.95	98.48	98.36	98.19	**99.75**	98.72	96.26	99.25	98.43	98.05
	SP (%)	98.24	97.80	93.97	97.26	**99.80**	98.26	87.70	95.99	93.88	97.10
	F1_score_	0.99	0.98	0.96	0.98	**1.00**	0.99	0.92	0.98	0.96	0.98
	MCC	0.97	0.96	0.92	0.95	**1.00**	0.97	0.84	0.95	0.92	0.95
*P. trichocarpa*	ACC (%)	98.78	97.15	95.55	97.03	96.94	**99.81**	90.80	97.49	95.47	97.16
	SN (%)	98.38	98.15	97.68	97.46	97.05	**99.79**	93.22	99.36	98.45	97.69
	SP (%)	99.19	96.15	93.42	96.59	96.82	**99.84**	88.39	95.62	92.49	96.62
	F1_score_	0.99	0.97	0.96	0.97	0.97	**1.00**	0.91	0.98	0.96	0.97
	MCC	0.98	0.94	0.91	0.94	0.94	**1.00**	0.82	0.95	0.91	0.94
*S. moellendorffii*	ACC (%)	97.68	97.10	96.38	95.39	96.24	96.92	**99.09**	95.00	94.03	95.62
	SN (%)	98.95	98.26	98.67	98.22	98.77	98.65	**99.48**	98.45	98.25	97.34
	SP (%)	96.40	95.94	94.10	92.56	93.71	95.19	**98.70**	91.55	89.82	93.90
	F1_score_	0.98	0.97	0.97	0.96	0.96	0.97	**0.99**	0.95	0.94	0.96
	MCC	0.95	0.94	0.93	0.91	0.93	0.94	**0.98**	0.90	0.88	0.91
*G. sulphuraria*	ACC (%)	96.58	96.61	94.47	94.25	95.68	97.13	89.30	**99.41**	94.92	94.86
	SN (%)	95.97	95.94	96.02	94.37	95.14	96.90	89.78	**99.57**	96.22	94.70
	SP (%)	97.18	97.28	92.92	94.13	96.22	97.36	88.83	**99.25**	93.62	95.03
	F1_score_	0.97	0.97	0.95	0.94	0.96	0.97	0.89	**0.99**	0.95	0.95
	MCC	0.93	0.93	0.89	0.89	0.91	0.94	0.79	**0.99**	0.90	0.90
*T. aestivum*	ACC (%)	97.71	97.42	93.83	95.94	97.32	97.77	90.70	98.48	**99.75**	97.01
	SN (%)	98.38	97.39	98.07	97.91	98.37	98.70	93.30	99.04	**99.72**	97.75
	SP (%)	97.03	97.46	89.58	93.96	96.27	96.85	88.09	97.91	**99.79**	96.27
	F1_score_	0.98	0.97	0.94	0.96	0.97	0.98	0.91	0.99	**1.00**	0.97
	MCC	0.95	0.95	0.88	0.92	0.95	0.96	0.82	0.97	**1.00**	0.94
*S. lycopersicum*	ACC (%)	98.57	97.80	96.23	97.08	97.35	98.63	94.30	97.06	95.76	**99.53**
	SN (%)	98.63	98.74	98.13	97.46	98.75	98.63	95.78	99.23	99.15	**99.44**
	SP (%)	98.51	96.87	94.34	96.70	95.96	98.63	92.83	94.39	92.37	**99.62**
	F1_score_	0.99	0.98	0.96	0.97	0.97	0.99	0.94	0.97	0.96	**1.00**
	MCC	0.97	0.96	0.93	0.94	0.95	0.97	0.89	0.94	0.92	**0.99**

Table 7 presents an overview of the generalization performance of LMFE across ten distinct plant species. The evaluation encompasses a range of metrics, including ACC, SN, SP, F1_score_, and MCC. The table reveals that the diagonal values are typically higher than the off-diagonal values, signifying that LMFE achieves better results when tested within the same species it was trained on. For instance, the ACC for *P. trichocarpa* is notably high at 99.81%, while *S. lycopersicum* exhibits an impressive SN of 99.44%. These figures underscore LMFE’s proficiency in correctly identifying positive samples within their respective species. In terms of cross-species validation, the LMFE trained on the *A. thaliana* demonstrates superior performance across all tested species, with a highest accuracy of 99.09% on *P. trichocarpa*. Other species, such as *V. radiata* and *Z. mays*, also show commendable performances, with accuracies of 98.46% and 98.89%, respectively. This suggests strong generalization capability across different plant species. It is worth noting that LMFE trained on G. sulphuraria performed relatively poorly when validated on *S. moellendorffii*, with an accuracy of 89.3% and an MCC of 0.79. Similarly, LMFE trained on *T. aestivum* achieved an accuracy of 90.7% on *S. moellendorffii*, also indicating potential for improvement in these cases. Overall, the experimental outcomes indicate that LMFE exhibits strong recognition capabilities across various plant species, particularly in terms of SN and SP. The metrics ACC, SN, and SP generally surpass 90%, while the F1_score_ typically exceeds 0.90, demonstrating an effective balance between ACC and SN. Despite some variations in performance across species, the overall generalization ability remains robust. The highest values in the table have been bolded.

**Table 8:** The performance of LMFE on unbalanced datasets.

Species	ACC (%)		Recall (%)		F1_score_	
	EXP NO-SMOTE	EXP WITH-SMOTE	EXP NO-SMOTE	EXP WITH-SMOTE	EXP NO-SMOTE	EXP WITH-SMOTE
*G. max*	95.66	**99.** **04**	93.56	**99.40**	0.97	**0.99**
*M. domestica*	79.84	**99.6** **2**	**100.00**	99.84	0.73	**1.00**
*A. officinalis*	90.22	**99.** **50**	86.77	**99.2** **6**	0.93	**1** **.** **00**
*L. angustifolius*	82.75	**99.** **46**	**100.00**	99.02	0.75	**1.00**

Table 8 demonstrates that on unbalanced datasets, LMFE attained an average ACC of 99.41%, representing a 12.29% improvement over traditional methods (average ACC of 87.12%) after applying SMOTE. The comparative experimental results indicate that the accuracy, recall, and F1_score_ in EXP WITH-SMOTE are better than those in EXP NO-SMOTE, indicating that the introduction of the SMOTE method effectively improves LMFE’s recognition ability for minority class samples, thereby enhancing the overall performance of LMFE. This result further verifies the effectiveness of SMOTE in addressing imbalanced datasets, underscoring its significant application value in bioinformatics and data mining. The highest values in the table have been bolded.

**Table 9:** The performance comparison between LMFE and state-of-the-art methods.

Species	CPC2	PLEKv2	LGC	CNCI	PlncRNA-HDeep	LMFE
*V. angularis*	96.20	87.22	96.05	81.55	94.63	**98.** **8** **5**
*S. indicum*	96.06	88.37	94.53	85.78	**99** **.** **12**	98.69
*B. distachyon*	94.58	85.75	93.95	84.72	96.33	**99.** **05**
*M. acuminat* *a*	96.85	89.04	95.71	85.79	**99** **.** **60**	99.00
*M. polymorpha*	92.63	86.67	92.63	78.87	76.88	**99.2** **1**
*N. colorata*	91.64	85.98	88.71	76.64	94.40	**97.** **33**

Table 9 confirms the ACC of six state-of-the-art methods for plant lncRNA. LMFE consistently achieves the highest ACC across all species, ranging from 97.14% to 99.21%, demonstrating its superior and stable performance. PlncRNA-HDeep shows high accuracy in most species, peaking at 99.6% in *M. acuminata* and 99.12% in *S. indicum*, but drops significantly to 76.88% in *M. polymorpha*, indicating variability. CPC2 and LGC perform reliably, with CPC2 ranging from 91.64% (*N. colorata*) to 96.85% (*M. acuminata*) and LGC from 88.71% (*N. colorata*) to 96.05% (*V. angularis*), though both are generally outperformed by LMFE. PLEKv2 maintains moderate accuracy, varying from 85.75% (*B. distachyon*) to 89.04% (*M. acuminata*), while CNCI consistently ranks lowest, with ACC values between 76.64% (*N. colorata*) and 85.79% (*M. acuminata*), highlighting its limited effectiveness in this comparison. The highest values in the table have been bolded.

## Data Availability

The source code and datasets for the LMFE project are hosted on GitHub and can be accessed at https://github.com/ben-mpu/LMFE, accessed on 24 March 2025.

## Supplementary Materials

The following supporting information can be downloaded at https://www.mdpi.com/article/10.3390/genes16040424/s1, Figures S1–S9: Performance comparisons of LMFE trained on different species (*V. radiata*, *Z. mays*, *S. bicolor*, *O. sativa*, *P. trichocarpa*, *S. moellendorffii*, *G. sulphuraria*, *T. aestivum*, *S. lycopersicum*) with other species. Each figure includes: (a) precision and recall. (b) ROC curves and AUC. Figures S10–S12: Confusion matrices for *M. domestica*, *A. officinalis*, and *L. angustifolus*. Figures S13–S17: Performance comparisons on *S. indicum*, *B. distachyon*, *M. acuminata*, *M. polymorpha*, and *N. colorata*. Each figure includes: (A) ROC curve comparison. (B) Precision, recall, and F1_score_ comparison. Table S1: Details of the benchmark dataset. Table S2: Details of the independent dataset. Table S3: Details of the unbalanced dataset. Table S4: Performance details of redundant features. Table S5: Operational methods of benchmark tools for plant lncRNA prediction.
