# Supplementary material for: LMFE: A Novel Method for Predicting Plant LncRNA Based on Multi-Feature Fusion and Ensemble Learning
Source: Genes (Basel). 2025 Mar 31;16(4):424. doi: 10.3390/genes16040424 (PMC12026654; doi:10.3390/genes16040424)
Supplement: Supplementary file 1 [file genes-16-00424-s001.zip › genes-3513464-supplementary.pdf]

Supplementary Materials Table S1. Details of the benchmark dataset.

Species	Downloaded Data		Total downloaded data	Processed Dataset		Total Processed data
	Positive Data	Negative Data		Positive Data	Negative Data	
A. thaliana	6775	48,321	55,096	6775	6775	13,550
V. radiata	5605	23,181	28,786	4600	4600	9200
Z. mays	11,572	72,539	84,111	11,572	11,572	23,144
S. bicolor	5428	47,110	52,538	5400	5400	10,800
O. sativa	6022	40,745	46,767	6003	6003	12,006
P. trichocarpa	5616	52,400	58,016	5615	5615	11,230
S. moellendorffii	2317	34,825	37,142	2300	2300	4600
G. sulphuraria	1896	7174	9070	1870	1870	3740
T. aestivum	15,321	86,669	101,990	6500	6500	13,000
S. lycopersicum	3407	34429	37836	3377	3377	6754
Total	63,959	447,393	511,352	54,012	54,012	108,024

Supplementary Materials Table S2. Details of the independent dataset.

Species	Download Dataset		Total Downloaded	Processed Dataset		Total Processed data
	Positive Data	Negative Data		Positive Data	Negative Data	
V. angularis	2103	33,860	35,963	2000	2000	4000
S. indicum	3516	25,172	28,688	3400	3400	6800
B. distachyon	3328	52,972	56,300	3000	3000	6000
M. acuminate	5717	45,589	51,306	5600	5600	11,200
M. polymorpha	1225	24,674	25,899	1200	1200	2400
N. colorata	2131	28,438	30,569	2100	2100	4200
Total	18,020	210,705	228,725	17,300	17,300	34,600

Supplementary Materials Table S3. Details of the unbalanced dataset.

Species	Download Data		Total downloaded	Processed Data		Total Processed data
	Positive Data	Negative Data		Positive Data	Negative Data	
G. max	4275	88,412	92,687	4000	2000	6000
M. domestica	5894	40,624	46,518	2000	5500	7500
A. officinalis	8957	24,141	33,098	6500	2300	8800
L. angustifolius	3652	33,083	36,735	1700	4800	6500
Total	22,778	186,260	209,038	14,200	14,600	28,800

Supplementary Materials Table S4. Performance details of redundant fe atures.

Features	Recall (%)	SN (%)	SP (%)	MCC	F1score
Dreduced	97.26	97.26	95.33	0.93	0.96
Dnum_unpaired_bases	97.54	97.54	95.35	0.93	0.97
Dnum_gc_pairs	97.53	97.53	95.11	0.93	0.96
Dgcc	97.26	97.26	95.36	0.93	0.96
Dauu	97.12	97.12	95.91	0.93	0.97
Dggc	97.65	97.65	95.50	0.93	0.97
Dorfs_length	98.80	98.80	98.44	0.97	0.99
Duua	98.65	98.65	98.90	0.98	0.99
Dgccontent	98.73	98.73	98.70	0.97	0.99
Dnum_au_pairs	98.78	98.78	98.55	0.97	0.99
Dcg	98.62	98.62	98.99	0.98	0.99
Dgcg	99.07	99.07	98.68	0.98	0.99
Daau	98.71	98.71	98.82	0.98	0.99
Dc	98.81	98.81	98.87	0.98	0.99
Dggg	98.82	98.82	98.41	0.97	0.99
Dseqlength	99.31	99.31	99.21	0.99	0.99
Daaa	99.62	99.62	98.58	0.98	0.99

Features	Recall (%)	SN (%)	SP (%)	MCC	F1score
Dnum.base_pairs	99.35	99.35	99.20	0.99	0.99
Dnum_internal_loops	99.36	99.36	99.16	0.99	0.99
Dgc	99.20	99.20	99.13	0.98	0.99
Dmfe	99.23	99.23	99.31	0.99	0.99
Da	99.35	99.35	99.07	0.98	0.99
Dcc	99.14	99.14	99.18	0.98	0.99
Daa	99.11	99.11	99.45	0.99	0.99
Datgcratio	99.31	99.31	98.95	0.98	0.99
Dg	99.19	99.19	99.31	0.99	0.99
Du	99.32	99.32	99.07	0.98	0.99
Dccg	99.24	99.24	99.34	0.99	0.99
Dorfs_count	99.21	99.21	99.40	0.99	0.99
Dcgg	99.65	99.65	98.52	0.98	0.99
Dau	99.18	99.18	99.50	0.99	0.99
Duau	99.38	99.38	99.42	0.99	0.99
Dccc	99.22	99.22	99.25	0.99	0.99
Dcgc	99.33	99.33	99.19	0.99	0.99
Duu	99.32	99.32	99.36	0.99	0.99
Duaa	99.33	99.33	99.32	0.99	0.99
Dua	99.22	99.22	99.41	0.99	0.99

Supplementary Materials Table S5. Operational methods of tools for plant lncRNA prediction.

Tool	Method	Access Link or Installation	Usage
CPC2	Provides a web server for online prediction.	http://cpc2.gao-lab.org/	Upload FASTA file on the server.
PLEKv2	A command-line tool for lncRNA prediction.	Download from sourceforge (https://sourceforge.net/projects/plek2/)	Command line: python PLEK2.py -i input.fa -m pl -o output.txt -t labels.txt.
LGC	Offers a web-based interface for submitting FASTA format files.	Web server: http://bigd.big.ac.cn/lgc/	Upload FASTA file on the web interface and download results.
CNCI	A command-line tool for lncRNA prediction.	Download from GitHub (https://github.com/www-bioinfo-org/CNCI)	Command line: python CNCI.py -f input.fa -o output
PlncRNA-HDeep	python script for lncRNA prediction.	Download from GitHub (https://github.com/kangzhai/PlncRNA-HDeep)	run python script using pyCharm..

Supplementary Materials Figure S1.

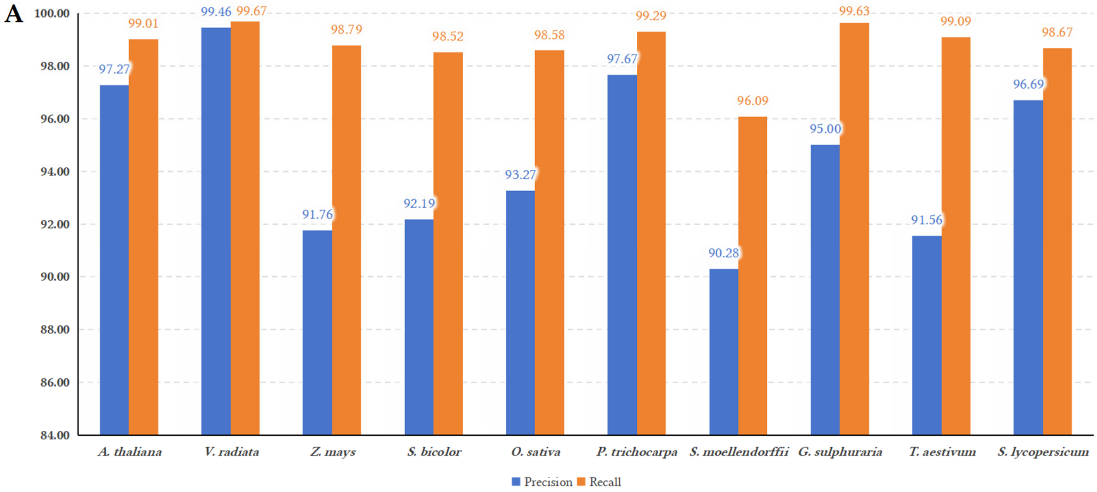

Figure S1 (A) compares the model's performance, trained on *V. radiata*, with other species using precision and recall.

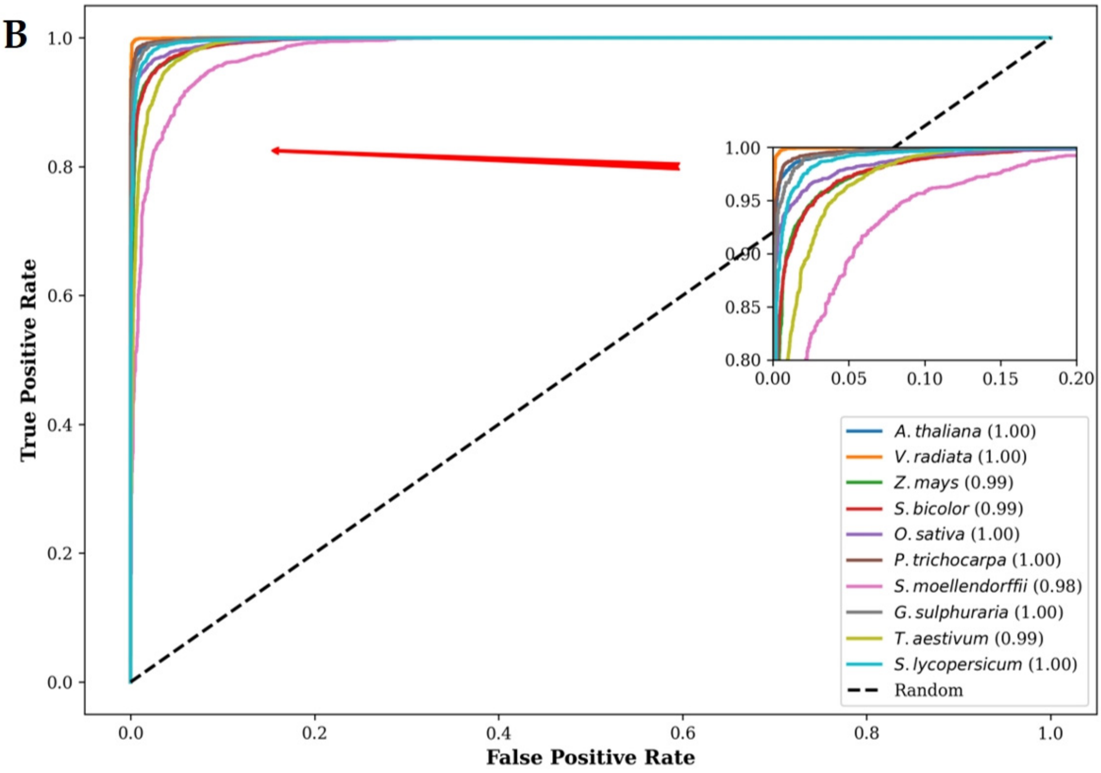

Figure S1 (B) compares the model's performance, trained on *V. radiata*, with other species using ROC curves and AUC value.

Supplementary Materials Figure S2.

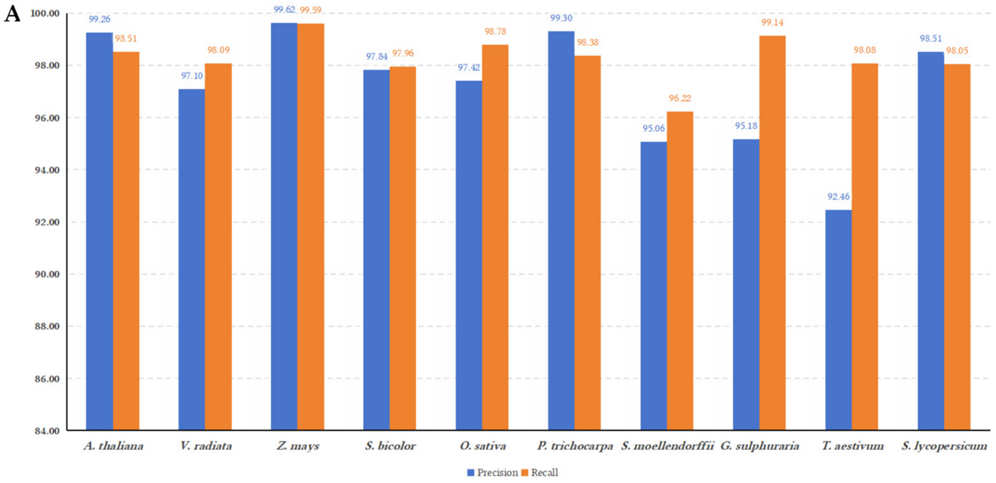

Figure S2 (A) compares the model's performance, trained on *Z. mays*, with other species using precision and recall.

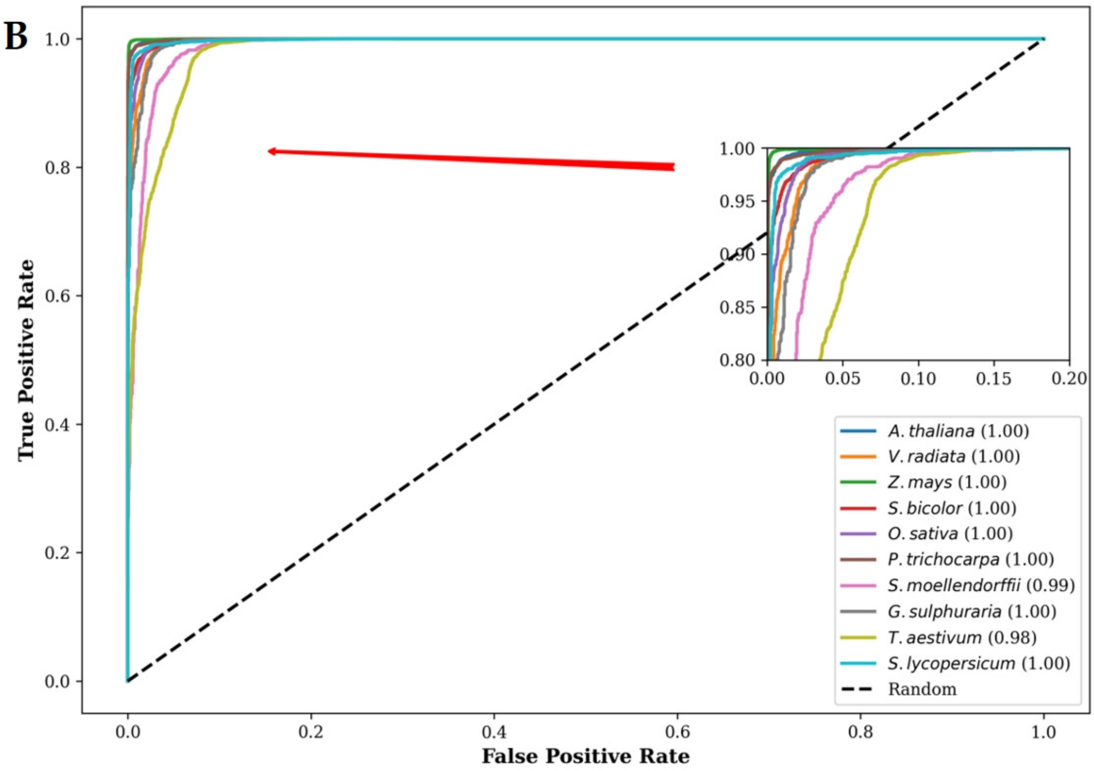

Figure S2 (B) compares the model's performance, trained on *Z. mays*, with other species using ROC curves and AUC value.

Supplementary Materials Figure S3.

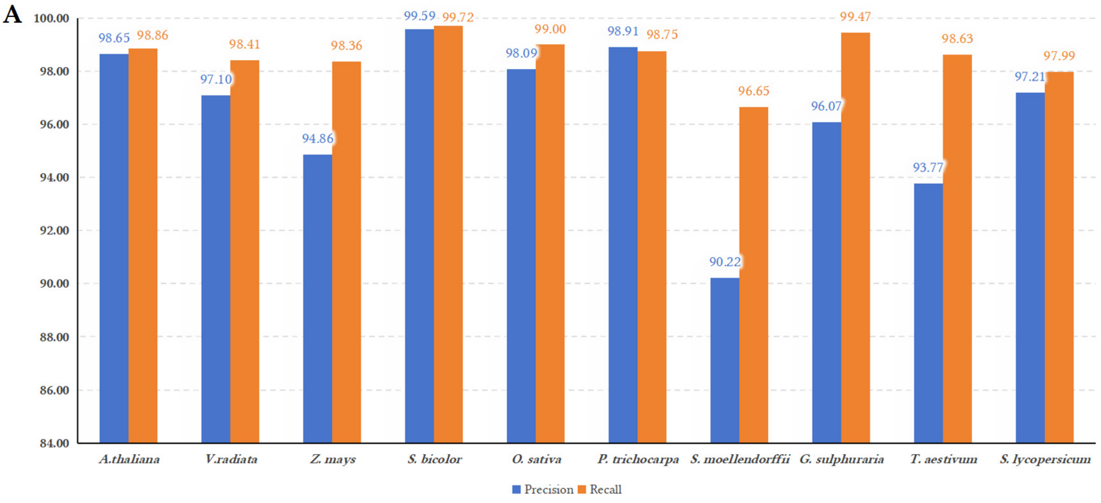

Figure S3 (A) compares the model's performance, trained on *S. bicolor*, with other species using precision and recall.

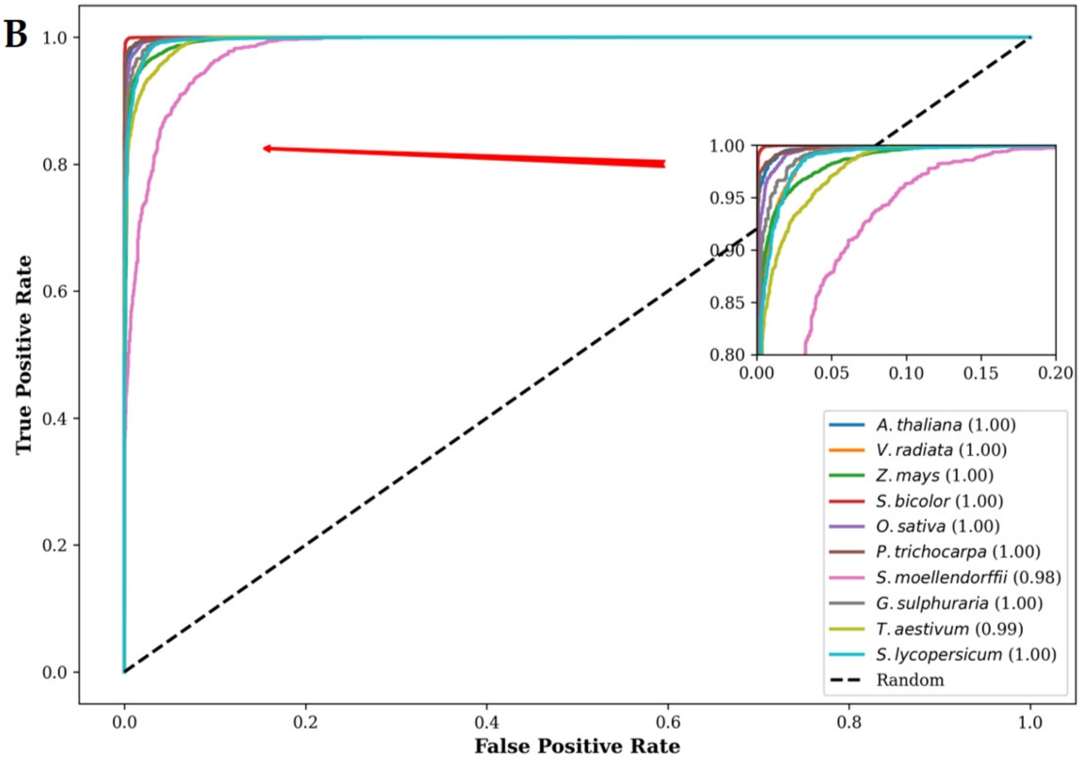

Figure S3 (B) compares the model's performance, trained on *S. bicolor*, with other species using ROC curves and AUC value.

Supplementary Materials Figure S4.

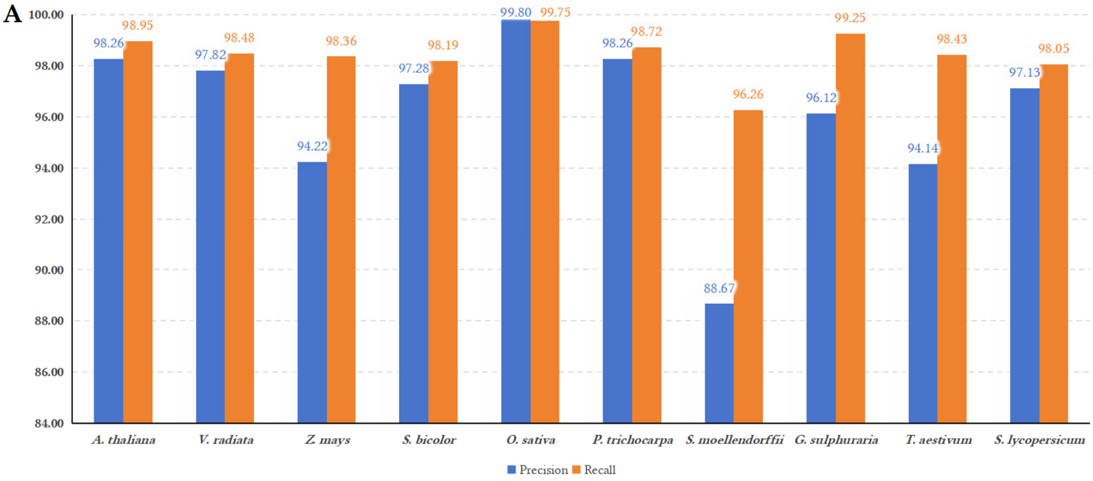

Figure S4 (A) compares the model's performance, trained on *O. sativa*, with other species using precision and recall.

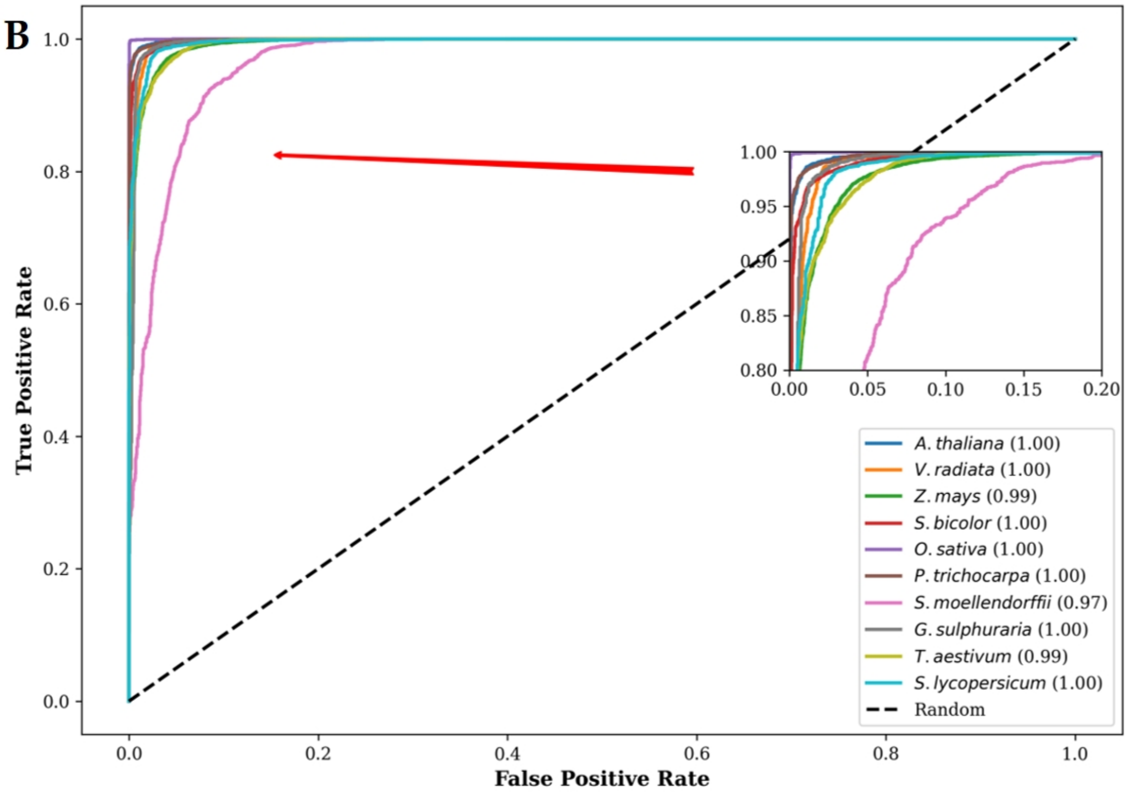

Figure S4 (B) compares the model's performance, trained on *O. sativa*, with other species using ROC curves and AUC value.

Supplementary Materials Figure S5.

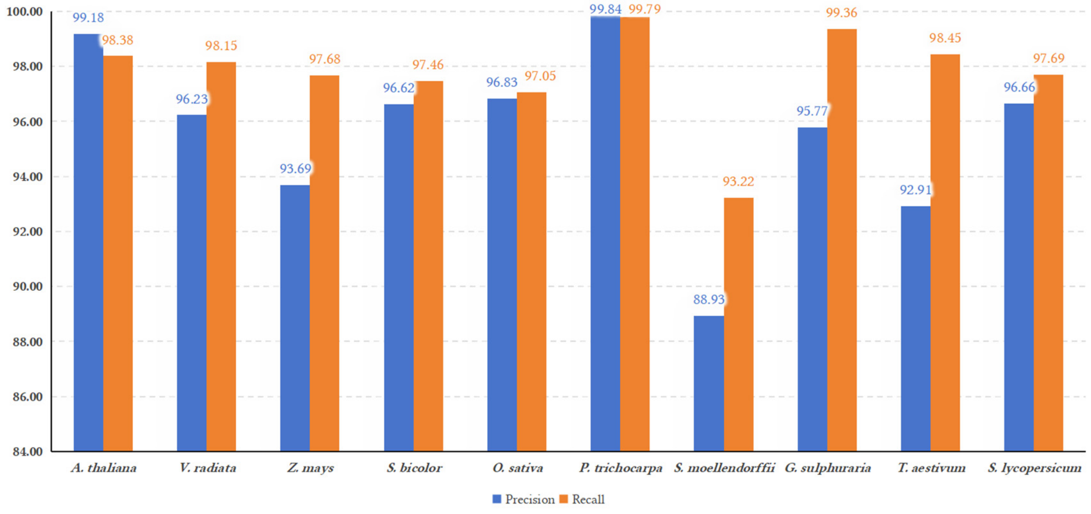

Figure S5 (A) compares the model's performance, trained on *P. trichocarpa*, with other species using precision and recall.

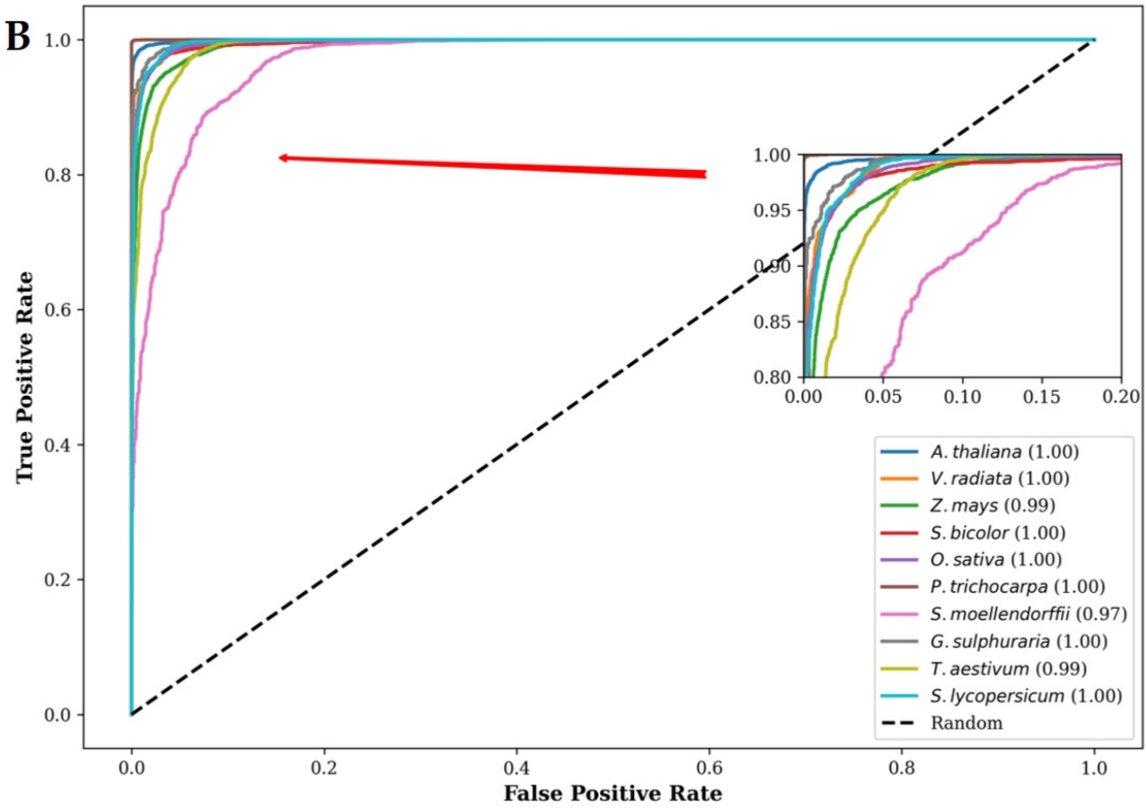

Figure S5 (B) compares the model's performance, trained on *P. trichocarpa*, with other species using ROC curves and AUC value.

Supplementary Materials Figure S6.

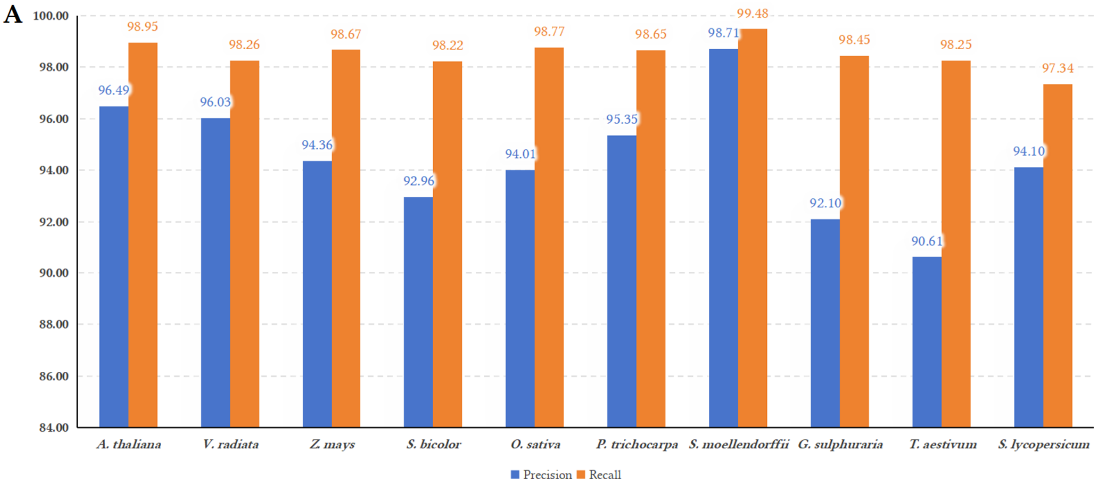

Figure S6 (A) compares the model's performance, trained on *S. moellendorffii*, with other species using precision and recall.

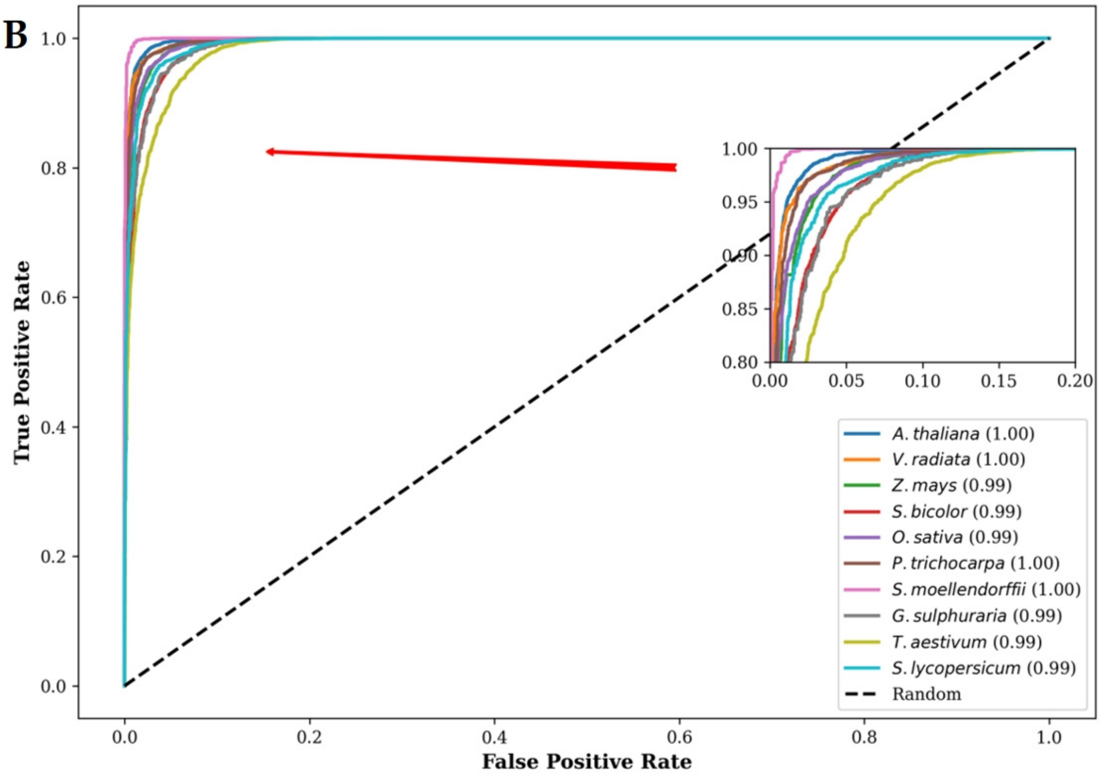

Figure S6 (B) compares the model's performance, trained on *S. moellendorffii*, with other species using ROC curves and AUC value.

Supplementary Materials Figure S7.

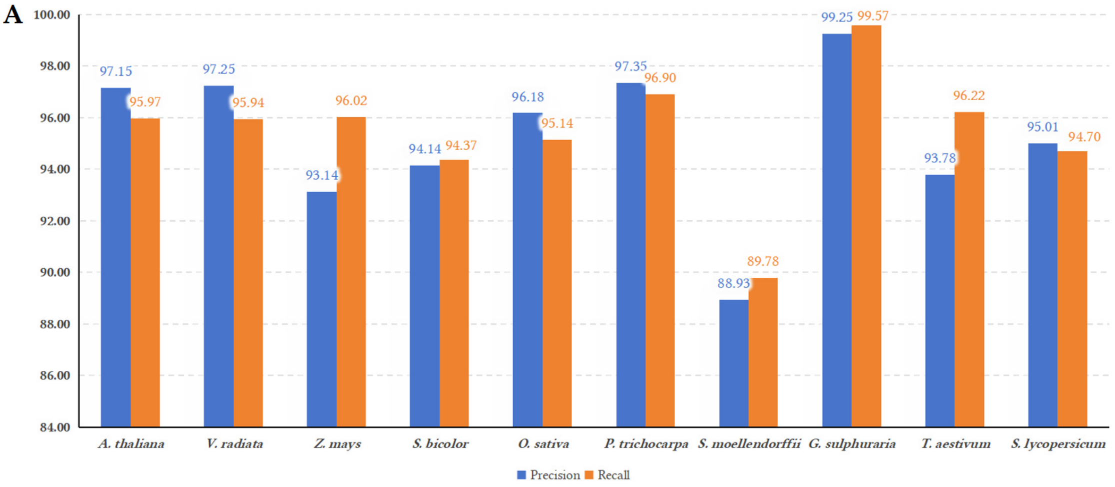

Figure S7 (A) compares the model's performance, trained on *G. sulphuraria*, with other species using precision and recall.

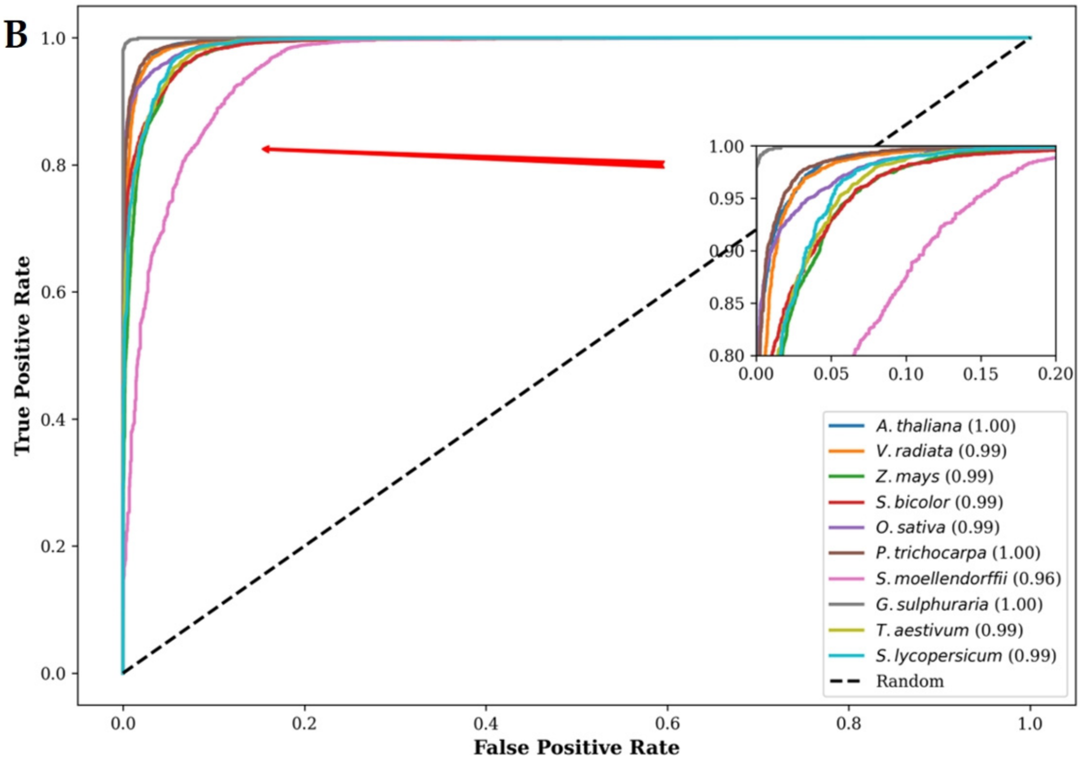

Figure S7 (B) compares the model's performance, trained on *G. sulphuraria*, with other species using ROC curves and AUC value.

Supplementary Materials Figure S8.

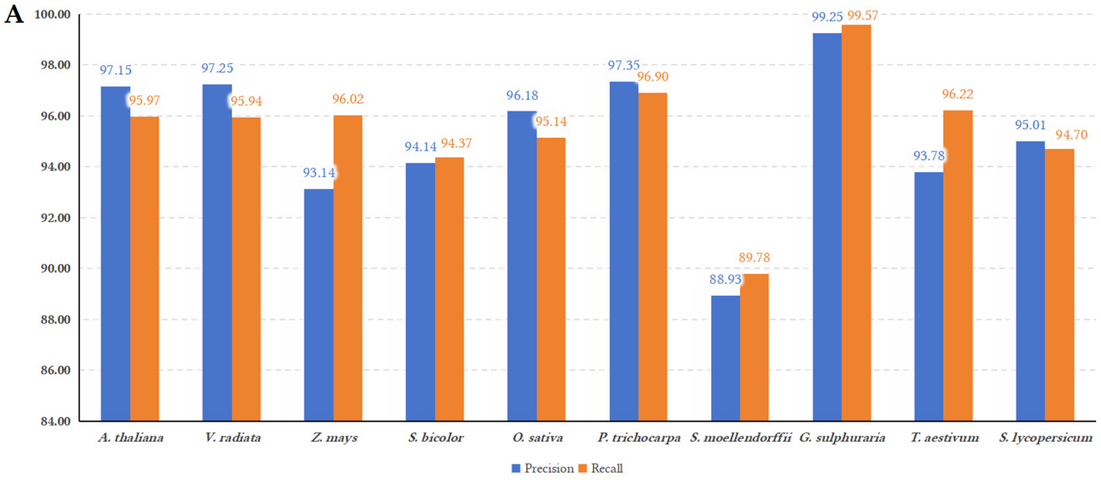

Figure S8 (A) compares the model's performance, trained on *T. aestivum*, with other species using precision and recall.

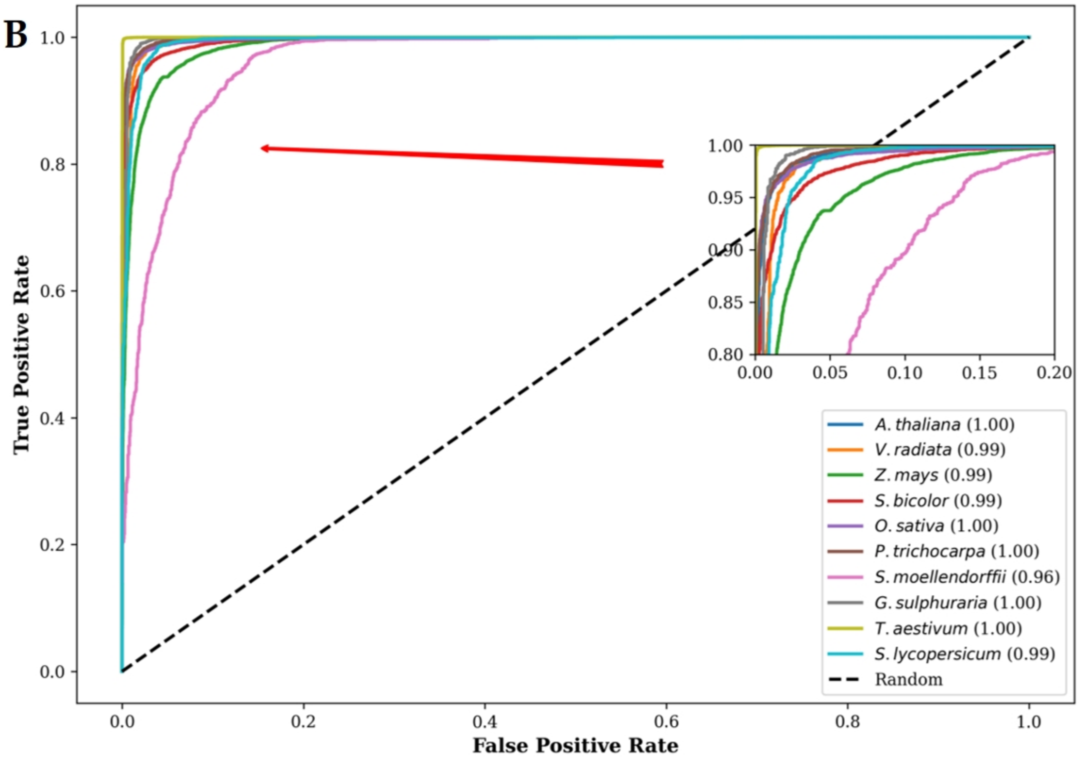

Figure S8 (B) compares the model's performance, trained on *T. aestivum*, with other species using ROC curves and AUC value.

Supplementary Materials Figure S9.

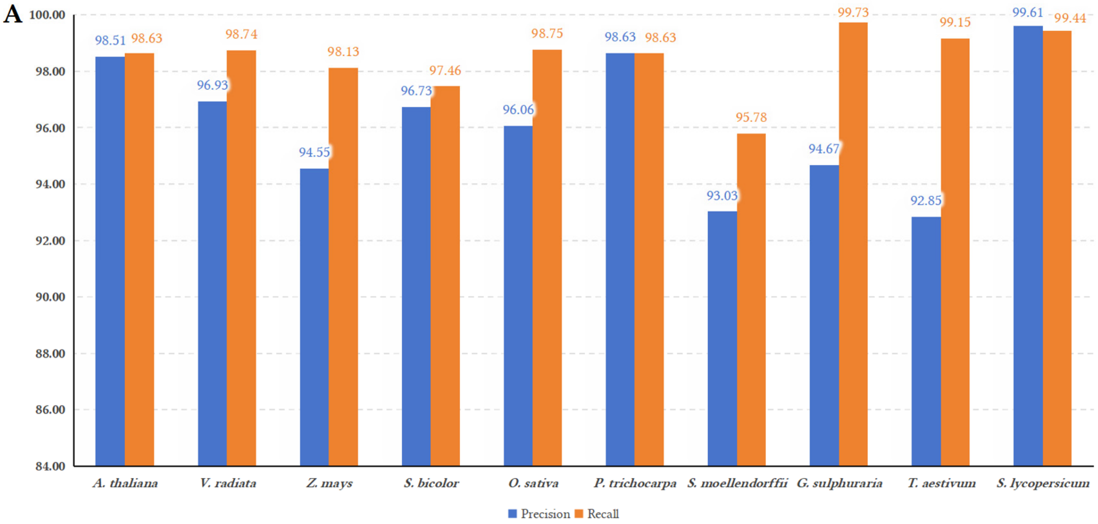

Figure S9 (A) compares the model's performance, trained on *S. lycopersicum*, with other species using precision and recall.

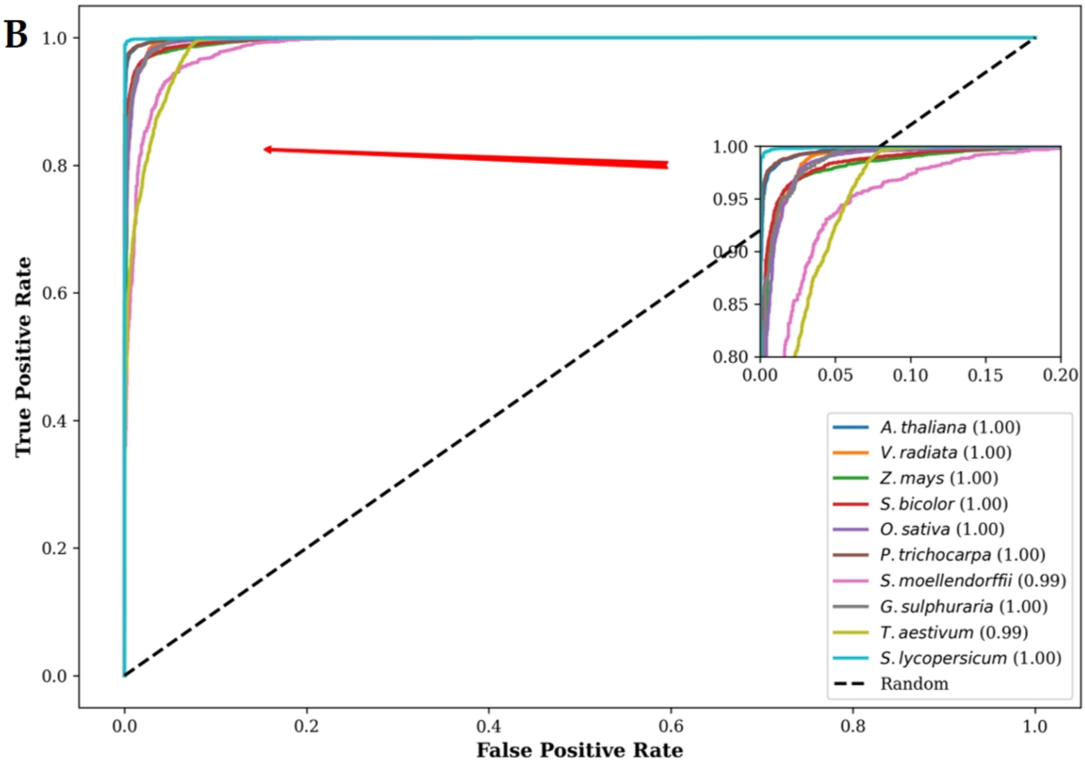

Figure S9 (B) compares the model's performance, trained on *S. lycopersicum*, with other species using ROC curves and AUC value.

Supplementary Materials Figure S10.

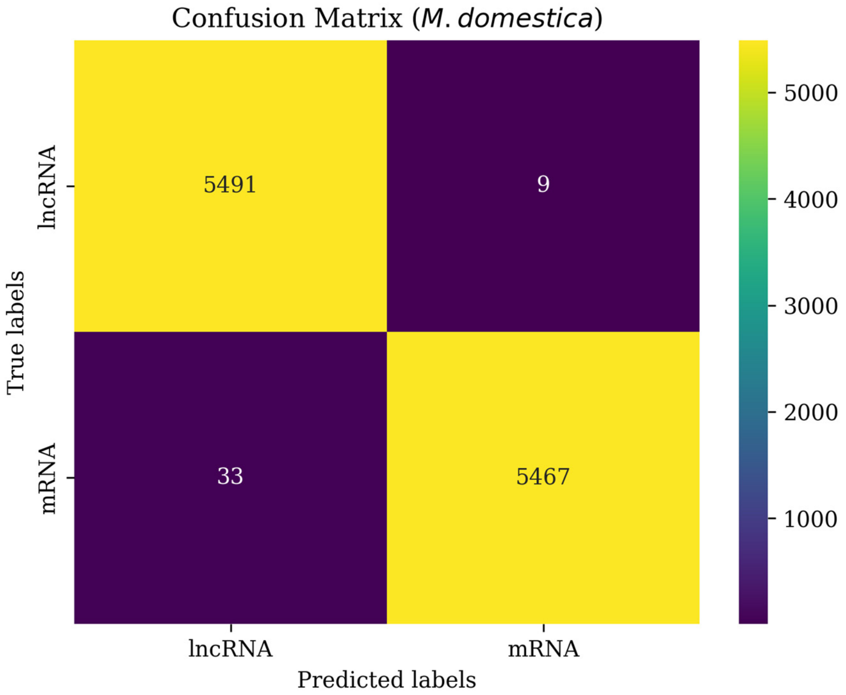

Figure S10 illustrates the confusion matrix on *M. domestica after applying SMOTE*.

Supplementary Materials Figure S11.

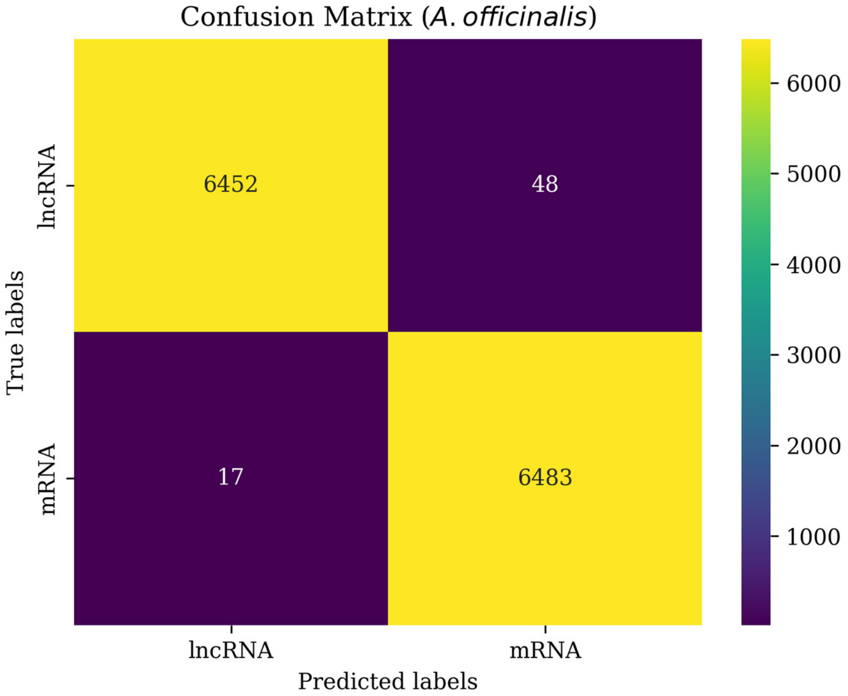

Figure S11 illustrates the confusion matrix on *A. officinalis after applying SMOTE*.

Supplementary Materials Figure S12.

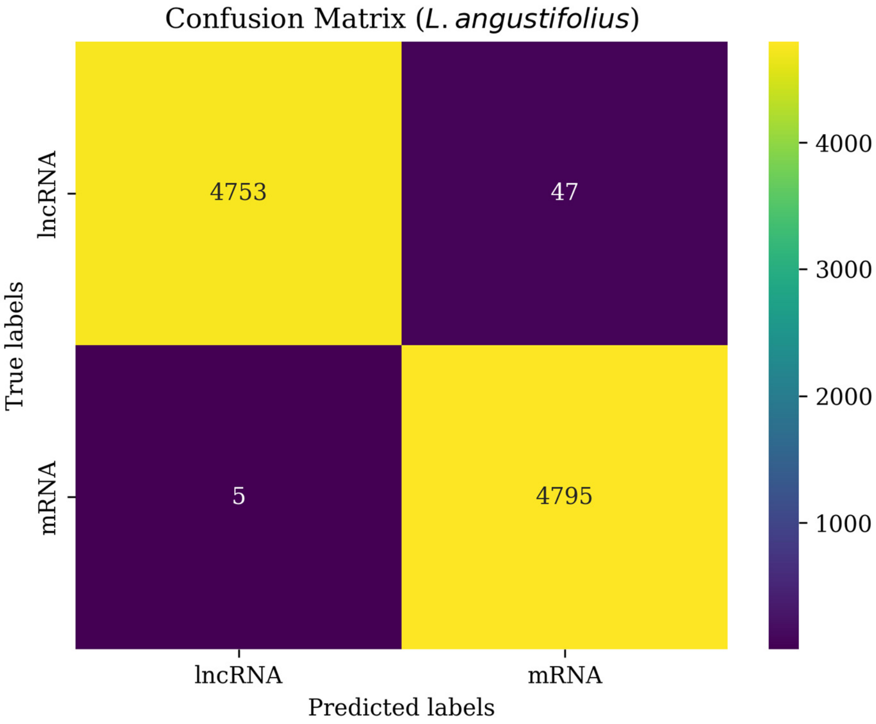

Figure S12 illustrates the confusion matrix on *L. angustifolius after applying SMOTE*.

Supplementary Materials Figure S13.

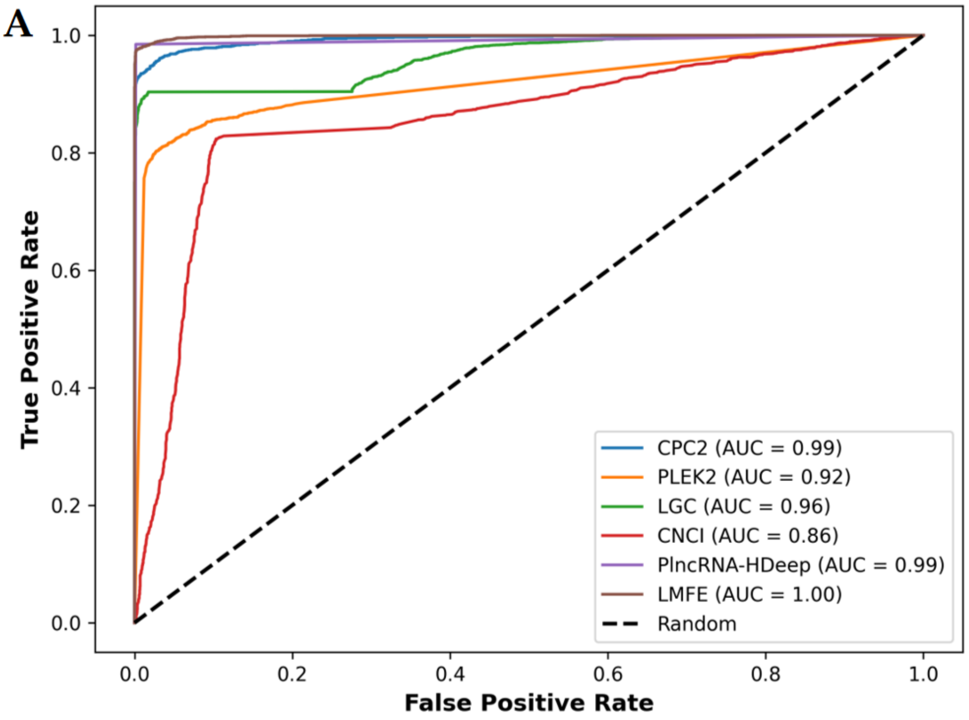

Figure S13 (A) illustrates ROC Curve Comparison on *S. indicum* dataset.

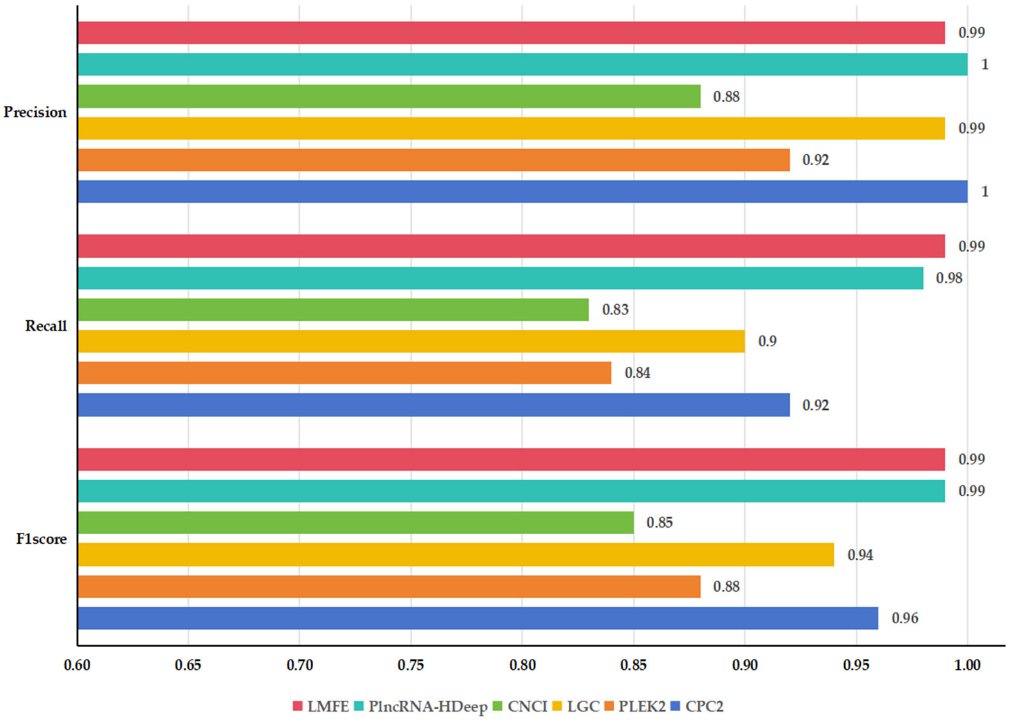

Figure S13 (B) illustrates Precision, Recall and F1score Comparison on *S. indicum* dataset.

Supplementary Materials Figure S14.

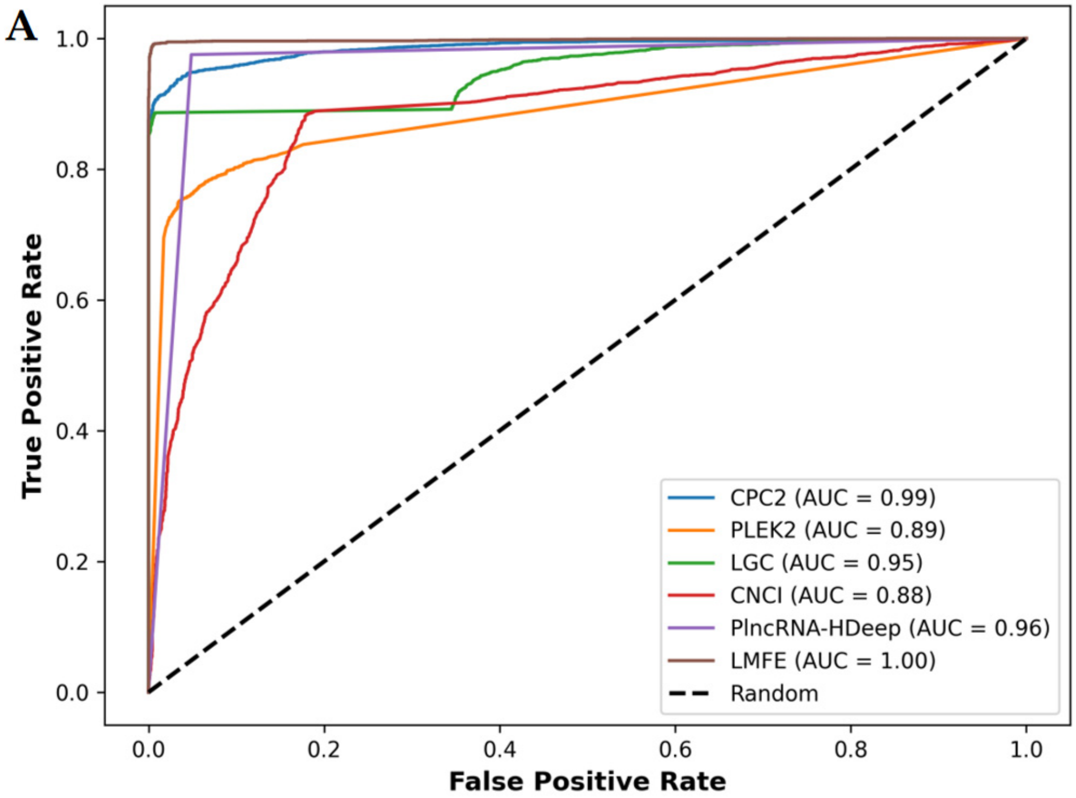

Figure S14 (A) illustrates ROC Curve Comparison on *B. distachyon* dataset.

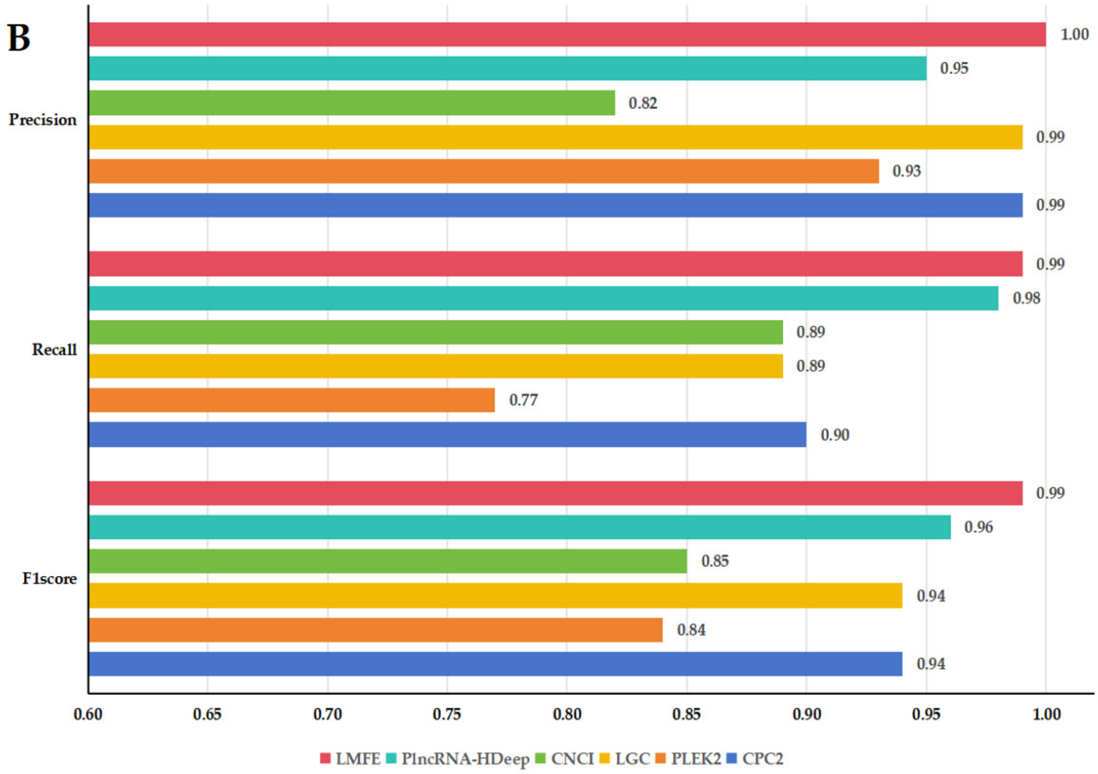

Figure S14 (B) illustrates Precision, Recall and F1score Comparison on *B. distachyon* dataset.

Supplementary Materials Figure S15.

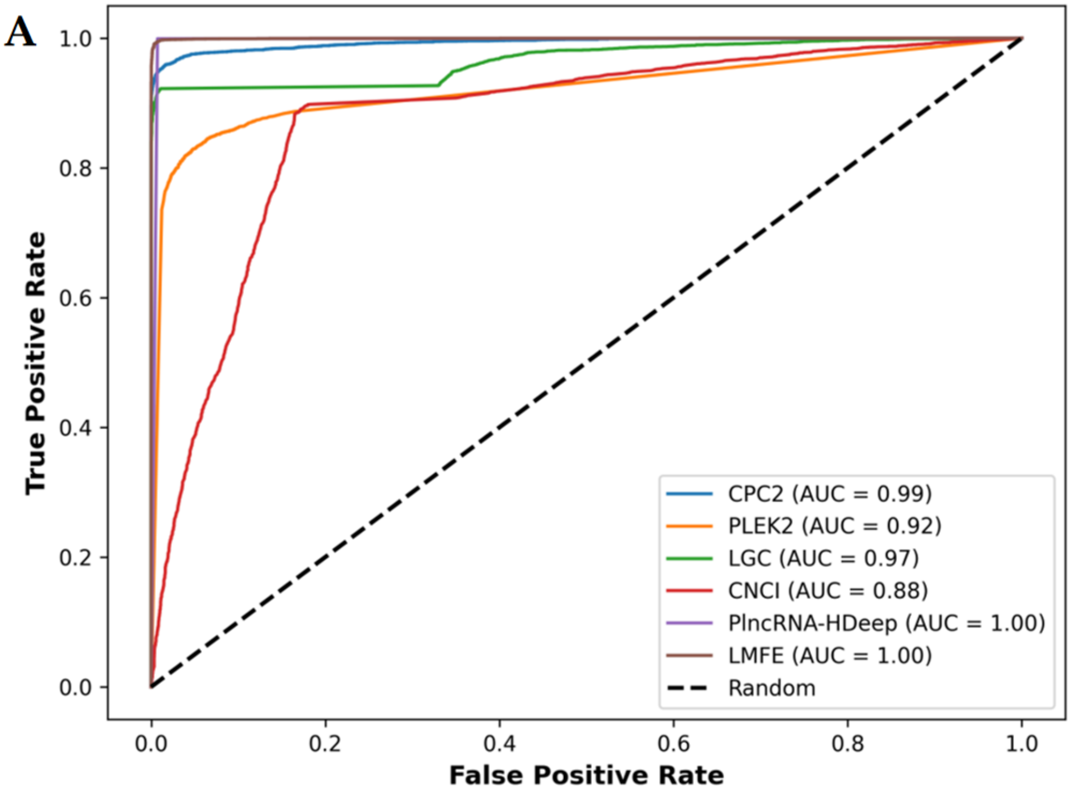

Figure S15 (A) illustrates ROC Curve Comparison on *M. acuminate* dataset.

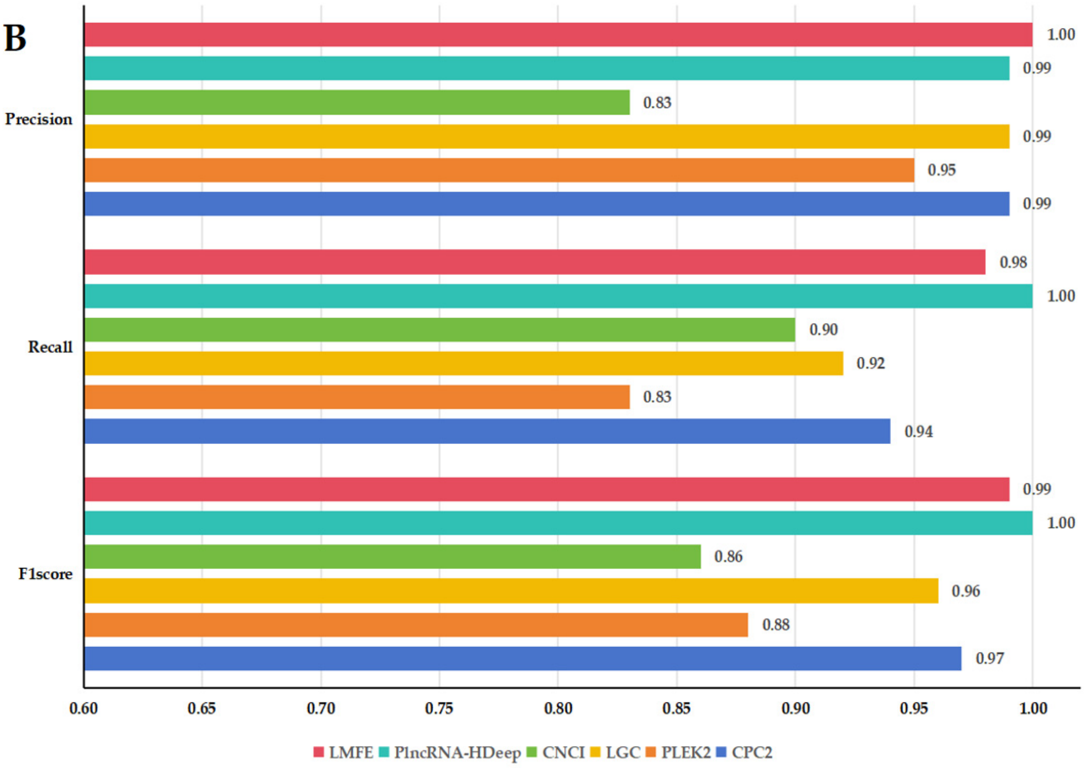

Figure S15 (B) illustrates Precision, Recall and F1score Comparison on *M. acuminate* dataset.

Supplementary Materials Figure S16.

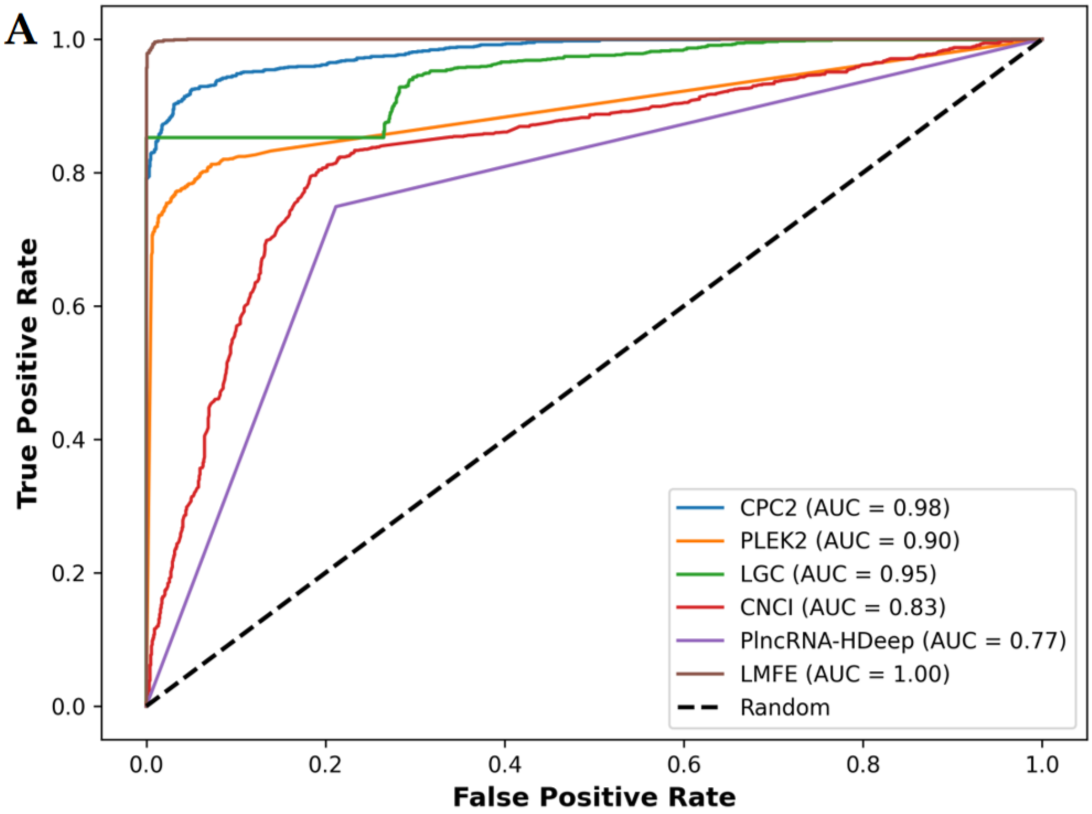

Figure S16 (A) illustrates ROC Curve Comparison on *M. polymorpha* dataset.

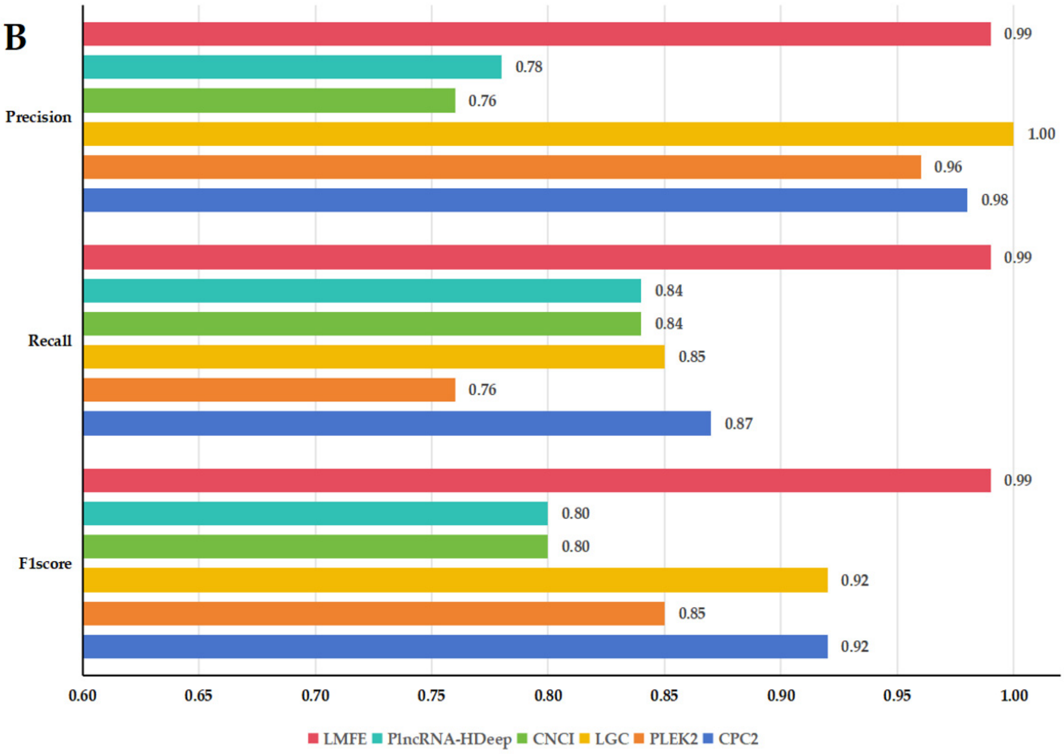

Figure S16 (B) illustrates Precision, Recall and F1score Comparison on *M. polymorpha* dataset.

Supplementary Materials Figure S17.

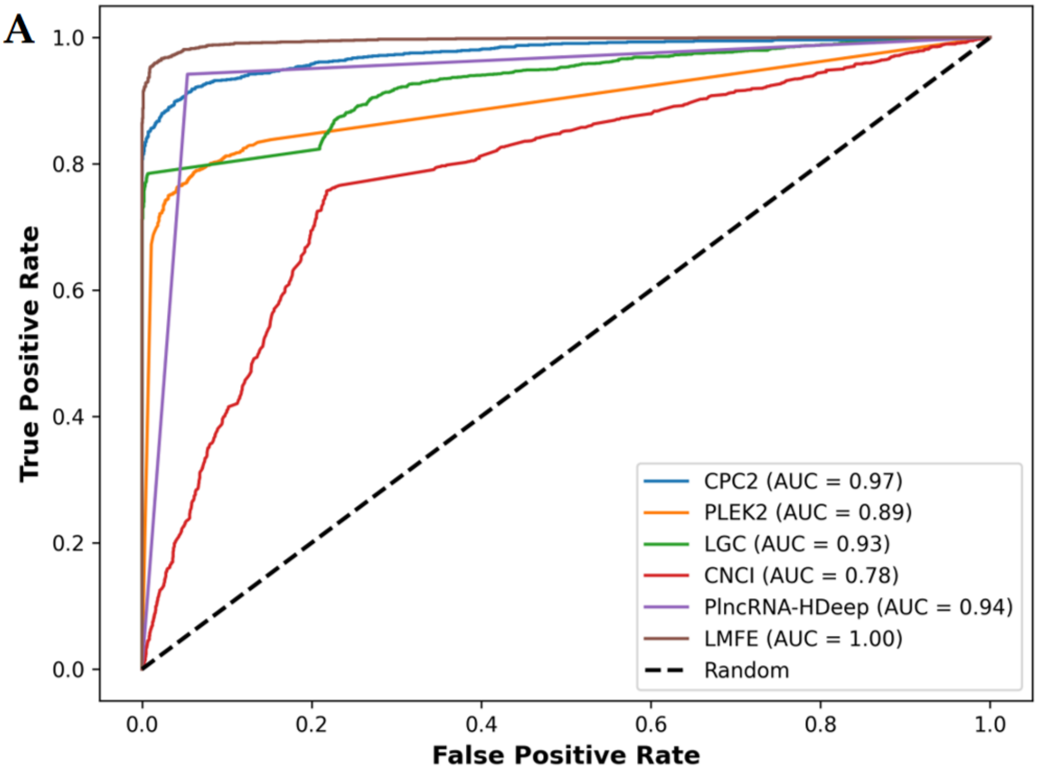

Figure S17 (A) illustrates ROC Curve Comparison on *N. colorata* dataset.

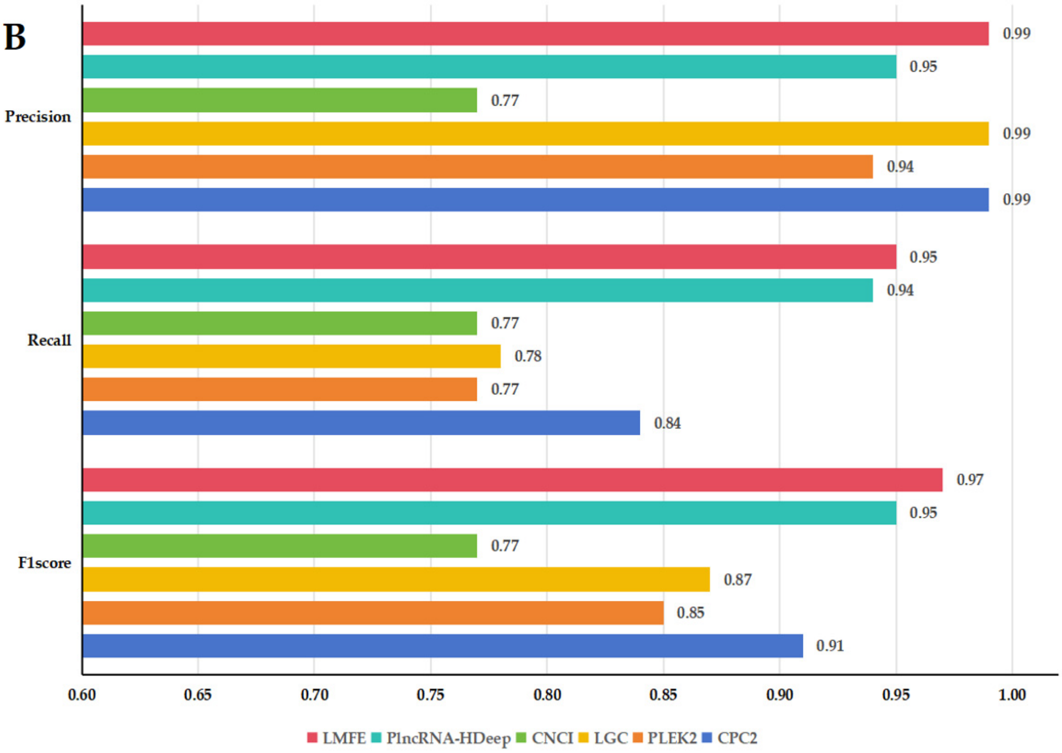

Figure S17 (B) illustrates Precision, Recall and F1score Comparison on *N. colorata* dataset.
